# Tracking the Spatial and Functional Dispersion of Vaccine-Related Canine Distemper Virus Genotypes: Insights from a Global Scoping Review

**DOI:** 10.3390/v17081045

**Published:** 2025-07-27

**Authors:** Mónica G. Candela, Adrian Wipf, Nieves Ortega, Ana Huertas-López, Carlos Martínez-Carrasco, Pedro Perez-Cutillas

**Affiliations:** 1 Animal Health Department, Faculty of Veterinary, University of Murcia, 30100 Murcia, Spain; adrianwm97@hotmail.com (A.W.); nortega@um.es (N.O.); cmcpleit@um.es (C.M.-C.); 2 Investigación Multidisciplinar en Ciencias Veterinarias (IMCivet), Universidad Católica de Murcia (UCAM), 303107 Murcia, Spain; ahuertas@ucam.edu; 3 SALUVET, Animal Health Department, Faculty of Veterinary, Complutense University of Madrid, 28040 Madrid, Spain; 4 Geography Department, Faculty of Humanities, University of Murcia, 30001 Murcia, Spain; pedrope@um.es

**Keywords:** America-1, CDV, *Morbillivirus canis*, multi-host pathogen, Rockborn-like, transmission, spread, spillover, surveillance, vaccines, vaccine genotypes

## Abstract

Canine morbillivirus (CDV), the cause of canine distemper, is a pathogen affecting many hosts. While modified live virus (MLV) vaccines are crucial for controlling the disease in dogs, cases of vaccine-related infections have been found in both domestic and wild animals. Specifically, the America-1 and Rockborn-like vaccine genotypes are concerning due to their spread and ability to transmit between different species. This study conducted a review and analysis of molecular detections of these strains in various carnivores (domestic, captive, synanthropic, and wild species). This study used a conceptual model considering host ecology and the domestic–wild interface to evaluate plausible transmission connections over time using Linear Directional Mean (LDM) and Weighted Mean Centre (WMC) methods. Statistical analyses examined the relationship between how likely a strain is to spread and factors like host type and vaccination status. The findings showed that the America-1 genotype spread in a more organised way, with domestic dogs being the main source and recipient, bridging different environments. Synanthropic mesocarnivores also played this same role, with less intensity. America-1 was most concentrated in the North Atlantic and Western Europe. In contrast, the Rockborn-like strain showed a more unpredictable and restricted spread, residual circulation from past use rather than ongoing spread. Species involved in vaccine-related infections often share characteristics like generalist behaviour, social living, and a preference for areas where domestic animals and wildlife interact. We did not find a general link between a host vaccination status and the likelihood of the strain spreading. The study emphasised the ongoing risk of vaccine-derived strains moving from domestic and synanthropic animals to vulnerable wild species, supporting the need for improved vaccination approaches. Mapping these plausible transmission routes can serve as a basis for targeted surveillance, not only of vaccine-derived strains, but of any other circulating genotype.

## 1. Introduction

*Morbillivirus canis,* also known as canine distemper virus (CDV), is a morbillivirus belonging to the *Paramyxoviridae* family that causes a highly contagious, acute, and fatal disease, canine distemper (CD) [1]. CDV can produce a fatal multisystemic disease affecting the immune, respiratory, gastrointestinal, and nervous systems. Infection commonly leads to lymphoid depletion, hyperkeratosis, interstitial pneumonia (often complicated by opportunistic bacterial infections), and encephalopathy, which may result in death [2,3,4,5,6]. Virions are highly contagious, transmitted through aerosolised nasal, oral, and ocular fluids, and their major route of entry for infection is through the respiratory system [7,8]. Because CDV is unstable in the environment, its survival and dispersion rely on susceptible hosts to continually infect and spread among animal populations [9].

CDV is a pathogen with worldwide distribution that mainly affects a wide range of wild and domestic species of the order Carnivora, but with continuous reports of CDV in other orders of terrestrial mammals [10]. Despite its broad range, domestic dogs remain the main reservoir host for CDV, although many wild carnivore species can also act as reservoirs and suffer CD [10,11,12]. In fact, species jump and spillover events from domestic dog reservoirs to wild carnivore species have led to high mortality outbreaks that represent a major conservation threat [13]. However, due to its wide host range, managing CDV in ecosystems with endangered species becomes a major challenge [14]. Since the 1950s, vaccination has been the primary strategy to control CDV in domestic dogs, historically considered the main host. Furthermore, the need to vaccinate wild species at risk remains controversial because there is a lack of a safe and effective vaccine for non-domestic species [15].

Vaccination stimulates both humoral and cellular responses through B and T lymphocytes, remaining the most effective way to prevent viral infections and their spread [16,17,18]. The immune response to CDV is long-lasting, potentially life-long [19,20], and the majority of commercial vaccines contain live modified CDV strains (named MLV or modified live virus vaccines) based on isolated CDV belonging to genotypes derived from the ancestral America-1 genotype [21]. The most commonly used strains for MLV vaccines, belonging to the America-1 genetic lineage, are Onderstepoort, Lederle, Snyder Hill, Convac, and CDV3. The first strain to be included in the CDV vaccine was Ondersteeport, isolated from foxes in 1939 in the USA and attenuated since the 1950s in avian cell lines [16]. Recently, given the genetic similarity of the vaccine strains described with genotypes classified as Asia-3 and detected in Asia, George et al. proposed the name Vaccine/Asia-3/North America-1 [22]. Another genetic lineage used to create MLV vaccines is the Rockborn strain. It was isolated in a domestic dog in Sweden in the 1950s and attenuated in dog kidney cell lines [16,23], and it is closer to the America-2 genotype, although Harde and Osterhaus [17] placed it in their sequencing with a 99% similarity in cluster together with Ondersteeport.

The strains used in MLV vaccines have frequently caused post-vaccination CD in both domestic dogs and wild carnivore species. Researchers around the world have also sequenced them in CD outbreaks affecting various wild host species [17,22,24,25,26]. In this regard, Martella et al. [16] state that both Rockborn-like CDV strains (derived from the original Rockborn strain but with mutations or recombinations) and Ondersteeport strains are spreading in the field and have undergone mutations as they have passed through a broad spectrum of CDV-susceptible hosts. Regarding the Rockborn strain, one of the most widely reported hypotheses attributes post-vaccination CD to the strain’s residual virulence. On the other hand, in addition to the reversion to virulence with the use of MLV vaccines, there is additional concern among researchers regarding CDV-susceptible wild host populations, specifically the potential of shedding of the vaccine virus into the ecosystem after causing post-vaccination CD. This is particularly relevant given documented cases of host species such as the Siberian weasel (*Mustela sibirica*), black-footed ferret (*Mustela nigripes*), and grey foxes (*Urocyon cinereoargenteus*), which eliminate the vaccine virus post-vaccination with MLV vaccines [27].

Once the vaccine strain becomes extra-animal, it can spread and remain in the ecosystem, and the phenomenon involving CDV vaccine-related strain persistence is called by Martella et al. [16] as animalisation. The possibility of a vaccine strain persisting in a susceptible host population is related to (i) special ecosystem conditions for CDV persistence [28] and (ii) the existence of a density of potential and susceptible hosts that ensures contact ratios [29] and therefore the transmission of CDV by direct contact [30]. Increasing anthropisation and urbanisation modify ecosystem conditions and facilitate the creation of ecotones in which new and faster epidemiological processes develop, especially when there are host scenarios involving wild and domestic synanthropic mesocarnivores [31,32].

### Assumptions and Objectives

This study predicates two key observations in wild and domestic carnivores: (i) the manifestation of post-vaccination CD disease (defined as the presence of CD signs appearing in species after vaccination with MLV vaccines) and (ii) the detection and sequencing of America-1 and Rockborn-like vaccine genotypes in unvaccinated animals (including both clinically affected animals and asymptomatic carriers). We therefore assume that these CDV strains have caused not only post-vaccination CD disease by cross-species transmission, but also the animalisation of CDV vaccine strains. Therefore, vaccine-related CD disease cases may be due to (i) the presence of residual virulence in the vaccine following MLV vaccination [16], (ii) virulence reversion after vaccine attenuation in MLV-vaccinated animals [33], and (iii) natural infection with America-1 or Rockborn-like “animalised” CDV strains that has adapted to a suitable host species [22,34]. With this approach, our objectives are:

1. To systematise the documented history of vaccine-related disease manifestations associated with CDV, specifying the genotype involved in each case whenever possible.

2. To represent and analyse the spatial distribution of epidemiological parameters associated with host species, incorporating their ecological and biological traits, with particular emphasis on synanthropic mesocarnivores as key spreading species at the urban–natural interface zones [28].

3. To identify spatial and temporal patterns in the evolution of vaccine-related CDV lineages, distinguishing the functional circulation of America-1 and Rockborn-like genotypes in their historical context of vaccine usage [16].

4. To assess the directional and geographical structure of potential viral dispersion routes through functional spatial analysis, enabling the detection of consistent patterns across genotypes, host groups, and levels of epidemiological plausibility.

## 2. Materials and Methods

The methodological framework for analysing the dispersion of CDV lineages applied in this study is based on an exploratory–descriptive approach to analyse the global geographical and temporal dispersion of the America-1 and Rockborn-like genotypes. One of the main objectives is to visualise and interpret spatial, temporal, ecological, and host-related distribution patterns, avoiding inferring evolutionary pathways or direct transmission chains, given the nature and limitations of the available data.

### 2.1. Scoping Review and Data Compilation

This scoping review and spatial meta-analysis were conducted according to the Preferred Reporting Items for Systematic Reviews and Meta-Analyses Extension for Scoping Reviews (PRISMA-Scr) statement [35]. Table S1 shows the PRISMA Scr checklist (Supplementary File S1). Four independent researchers performed the systematic search, the quality assessment, and the data extraction. Data were cross-checked and a senior researcher solved any disagreements. Everything related to systematic searches in bibliographic sources can be consulted at Archive.org (https://archive.org/details/osf-registrations-kndfv-v1, accessed on 24 July 2024) or through the DOI https://doi.org/10.17605/OSF.IO/KNDFV (accessed on 24 July 2024).

**Figure 1:**
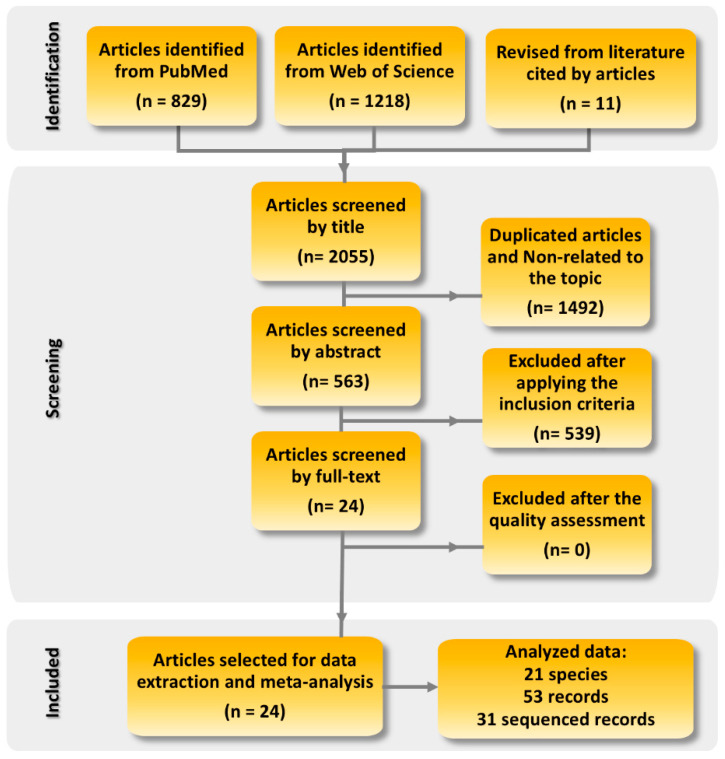
Article selection flowchart. Layers of information used to analyse data from articles that met all inclusion criteria established in this systematic review.

In brief, we performed a systematic search on the scientific databases Web of Science and PubMed, covering the period from 1985 to March 2025 (search date 30 March 2025). The following MeSH terms were employed: “(Canine distemper virus OR Morbillivirus canis OR Canine Morbillivirus) AND (Carnivore) AND (PCR OR Sequencing OR Phylogeny OR Phylogenetic OR Phylogenomic OR Genetic OR Characterisation OR Genetic lineage OR Haemaglutinin)”. The data were double-checked for all articles included in the qualitative synthesis. Initially, we found 2044 articles in the scientific databases, and the revision of their reference lists resulted in the inclusion of additional 11 articles (Figure 1). So, a total of 2055 articles were identified. Articles were reviewed at successive levels by title, abstract, and full text. After removing duplicated articles using a single EndNote file (EndNote Web 21, Clarivate Analytics, Philadelphia, PA, USA), we applied the following inclusion criteria:

1. Studies performed on domestic and/or wild carnivores.
2. Studies molecularly detecting CDV and subsequently sequencing the strain. CDV lineages were grouped following Panzera et al. [21] and Duque-Valencia et al. [12].
3. Studies that detect and sequence CDV classified within the America-1 and Rockborn-like genotypes.
4. Articles written in Spanish, Portuguese, and English.

Then, we assessed the quality of the 24 studies that met the inclusion criteria. The parameters considered for the quality assessment can be consulted at Wipf et al. [10]. We considered the lack of information regarding these parameters that could limit the reproducibility of the results as “high risk of bias,” and therefore these articles were excluded from the subsequent data extraction and spatial meta-analysis. According to these criteria, none of the 24 articles were considered to exhibit high risk of bias and therefore all of them were finally selected for data extraction and meta-analysis (Figure 1). Four researchers independently extracted the data by using a standardised Excel spreadsheet (Microsoft^®^ Excel for Microsoft 365 MSO, Version 2307, built 16.0.16626.20170). To analyse the data from the articles that met the criteria established for inclusion in this systematic review, we worked with different layers of information, including (Figure 1):

- Articles: Total number of articles reviewed (Table S2, Supplementary File S2).
- Species: Animal species analysed in the included articles.
- Records: Categorisation that classifies the different individual studies analysed in the articles reviewed. To work with and identify these individual studies, we considered the following criteria: (i) all the different species analysed in each revised article, (ii) the existence of sequencing of the two vaccine-related genotypes (America-1 and Rockborn-like), (iii) all the different geographical areas from which the host species originate.

We analysed a total of 21 variables related to (1) host species, (2) CDV genotypes considered, (3) date and geographical data, (4) host ecological and behavioural variables, (5) vaccination status, and (6) life status in relation to domestication (Table 1). Host species were classified into suborders Caniformia (Canidae, Mephitidae, Mustelidae, Procyonidae, Phocidae and Ursidae families) and Feliformia (Felidae, Herpestidae, Hyaenidae, and Viverridae families). Ecological and behavioural variables of the host species were incorporated, including their habitat type (closed, open, both), geographical distribution (restricted, worldwide), degree of ecological plasticity (specialist, generalist), social behaviour (solitary, gregarious), as well as the degree of interaction with human environments (i.e., the domestic–wildlife interface, including domestic dogs and captive, synanthropic, and elusive species).

**Table 1:** Analysed variables from the selected articles.

Data Category	Variable	Factor Label
Host species	Wild or domestic carnivore	Wild carnivore; domestic carnivore.
	Suborder	Caniformia; Feliformia
	Family	Canidae, Mephitidae, Mustelidae, Phocidae, Procyonidae, Ursidae, Felidae, Herpestidae, Hyanidae, Viverridae.
	Species	Common name.
	Species	Scientific name.
CDV linages	Genotype, following Panzera et al. [21] and Duque-Valencia et al. [12]	America-1, Rockborn-like (all vaccine strains classified within these genotypes).
Date and geographical procedence	Year of sampling	1988–2025
	Continent	Africa; America; Asia; Europe; Oceania.
	Country	
	Geographical region	
	Latitude coordinates (decimal degrees)	
	Longitude coordinates (decimal degrees)	
	Accuracy	Accurate; Approximate; National.
Host ecological and behavioral traits	Habitat type	Closed, open, both
	Geographical distribution	Restricted, worldwide.
	Degree of ecological plasticity	Specialist, generalist.
	Social behaviour	Solitary, gregarious.
	Contact with human environments	Yes, No.
Vaccination		Yes, No.
Host functional group. Life status in relation with domestication (Figure 2)	Domestic dog	*Canis lupus familiaris.*
	Captive carnivore	Zoo or farmed individuals.
	Synanthropic mesocarnivore	Species with periurban habits such as foxes, raccoons, and martens.
	Wild non-synanthropic carnivore	Those animals belonging Carnivora with limited or no human interface.

**Figure 2:**
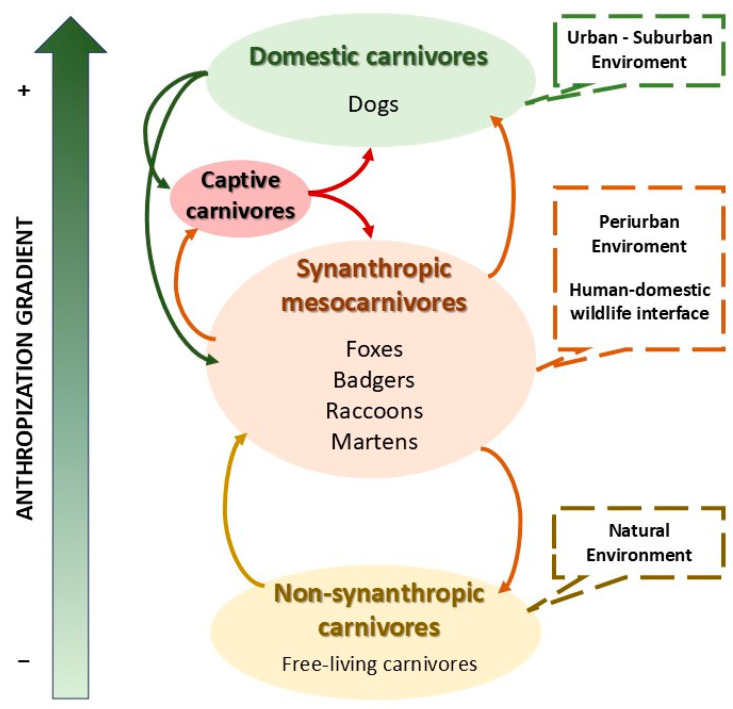
CDV infection flows considered for the analysis of plausible temporal connections based on the initial assumptions and hypotheses.

### 2.2. Epidemiological Modelling of Vaccine-Related CDV Dispersion

To identify transmission patterns associated with vaccine-related CDV genotypes, we developed a structured modelling approach. This second methodological block integrates both conceptual and analytical elements, focusing on the classification of host species, the temporal construction of transmission links, and the evaluation of plausibility for each potential connection. The aim was to generate a coherent framework from which patterns of viral circulation could be inferred, based not only on temporal and genetic criteria but also on biological and ecological patterns and spatial structure.

#### 2.2.1. Conceptual Model of Transmission Pathways

To establish a map of potential temporal connections between strains isolated from different hosts at different temporal and geographical stages, our working hypotheses for the spread of vaccine strains, once they were able to initiate CD disease processes and therefore transmission to new susceptible hosts, were based on the following criteria:

- Dogs, as the main host of CDV and a domestic species closely associated with humans, play a significant epidemiological role. They are highly susceptible to CDV and can become infected by any host species, regardless of the degree of interaction with human environments (anthropisation). Moreover, their high colonisation potential, generalist ecology, and the common practice of domestic dogs traveling extended distances with their owners allow them to disseminate the infection to areas far from its original focus.
- Wild carnivores are highly susceptible to CDV infection. In addition, the proliferation of synanthropic species, including mesocarnivores as the red fox, raccoon, skunk, and badger at the human–domestic–wildlife interface, boosts both intra- and interspecies contact rates. Increased densities of these species in anthropised areas facilitate the spread of directly transmitted viruses, such as CDV. In contrast, elusive carnivores with synanthropic habits often act as connectors between domestic and non-synanthropic wild reservoirs in their respective habitats.

In our study, these assumptions serve as the basis for determining the direction of CDV infection flows that we considered for the analysis of potential temporal and spatial connections (Figure 2). Our analysis specifically focused on information related to the following:

- Flow of CDV from domestic dogs to any carnivore species (both captive and wild), anywhere in the world.
- Transmission from any carnivore species (both captive and wild) globally to domestic dogs.
- Transmission among synanthropic mesocarnivores (specifically fox, raccoon, skunk and badger) and towards any other carnivore species (both captive and wild) within their shared geographical range.
- Transmission from any other elusive carnivore species that is neither generalist nor synanthropic (both captive and wild) towards synanthropic mesocarnivores (fox, raccoon, skunk and badger), within their shared geographical range.

Considering these hypotheses, we assumed that vaccine-related CDV strains, specifically those belonging to the America-1 and Rockborn-like genotypes, can give rise to limited but epidemiologically structured dispersion events in domestic and wild carnivore populations. We defined a conceptual framework to guide the selection and classification of transmission pathways based on the differentiation of three epidemiological scenarios along an anthropisation gradient: urban (considering areas where domestic dogs, cats, and other carnivore species under human management predominate), periurban (with generalist and synanthropic carnivore species typically described in human-modified environments), and natural environment (i.e., areas with minimal human-induced changes and where elusive, non-synanthropic wild species are mainly found). Within this framework, host species were grouped into four epidemiological host functional categories: (1) domestic dogs, (2) captive carnivores (e.g., zoo or farmed individuals), (3) synanthropic mesocarnivores (foxes, raccoons, badgers, and martens, as the top representative species), and (4) wild non-synanthropic carnivores (those elusive species with scarce adaptation to anthropised areas and consequently with limited interaction with humans and domestic carnivores). We used these groupings as a basis for evaluating the plausibility of CDV dispersion routes under vaccine-related scenarios and for assigning an ordinal potentiality score to each recorded or hypothesised connection (Figure 2).

#### 2.2.2. Generation and Filtering of Temporal Connection Data

Starting from a global review of genotype-specific CDV detections, we compiled two datasets of vaccine-related genotypes: 387 potential connections for America-1 and 61 for Rockborn-like genotypes. These connections were initially generated by pairing all temporally compatible records from the same genotype, assuming a unidirectional pathway from earlier to later detections. Each of these connections was then evaluated against the conceptual model described above and filtered using a set of predefined plausibility criteria. These included: (i) similarity between the biological traits and habitats occupied by the origin and destination carnivore species, (ii) presence or absence of a synanthropic interface, (iii) likely direction of inter-species transmission based on the epidemiological relevance described to date for each of the involved host species, and (iv) geographical spread feasibility, with an emphasis on cross-continental movement plausibility. We retained the resulting subsets, consisting of 246 epidemiologically plausible connections for America-1 and 55 for Rockborn-like, for all subsequent visual, statistical, and spatial assessments.

To further structure the spatial analysis, each retained connection was classified into one of seven levels of epidemiological plausibility based on a combination of ecological, temporal, and directional criteria. The classification relied on the functional group of the origin and destination hosts, considering their known epidemiological roles, ecological compatibility, and likelihood of sustained transmission under natural conditions. Level 1 represented connections between domestic dogs, followed by Level 2, which involved dogs and synanthropic mesocarnivores, and Level 3, typically between dogs and wild non-synanthropic carnivores. Higher levels (4 to 7) corresponded to combinations involving captive individuals, ecologically improbable pairings, or geographically scattered links. For Rockborn-like, only Levels 1, 3, 4, 5, and 7 were represented due to the limited diversity of host group combinations. This stratification allowed for a weighted representation of connections in spatial analyses, assigning higher influence to routes with stronger biological coherence. Additionally, all plausible connections respected a temporal sequence, whereby the origin host had to be detected prior to the destination host based on the reported date of infection or sampling. This assumption ensures that the epidemiological direction of the link is coherent, allowing for potential viral transmission from an earlier to a later case. This temporal criterion was essential in defining plausible routes, particularly in multi-host systems, and complements the ecological and functional criteria embedded in the potentiality level classification.

#### 2.2.3. Exploratory Data Analysis

Before the application of formal statistical testing, we conducted an exploratory characterisation of the retained datasets to assess the distribution and variability of key attributes. Variables such as host species, host functional group, continent, vaccination status, and captive/free-living status were summarised using frequency plots, cross-tabulations, and boxplots of dispersion potentiality. These visualisations helped identify dominant profiles, such as the prevalence of domestic dogs in America-1 connections, and guided the formulation of comparative hypotheses. This modelling approach provided the structural foundation for subsequent analyses. The host functional groupings, dispersion plausibility scores, and the filtered datasets of retained connections formed the analytical core of the study, enabling the identification of trends both through statistical testing and subsequently through spatial representations.

### 2.3. Statistical and Spatial Assessment of Transmission Patterns

Following the conceptual and structural modelling of vaccine-related CDV transmission routes, we applied a dual analytical approach to assess patterns emerging from the retained datasets. This approach combined statistical tests aimed at identifying host functional groups or geographical trends in the level of dispersion plausibility and spatial procedures designed to visualise potential transmission pathways across regions and ecosystems. We examined the distribution of transmission routes by host functional group (defined by ecological and biological characteristics of the host species and the degree of interaction with human environments) as well as their relation to estimated levels of dispersion potentiality. The analysis was structured around two main dimensions: (1) the role of host functional group (domestic dogs, captive carnivores, synanthropic mesocarnivores, and wild non-synanthropic carnivores) in shaping the plausibility of transmission and (2) the frequency and directionality of connections between host categories. While the statistical component explored associations between plausibility scores and variables such as host group, vaccination status, and geographic context, the spatial analysis focused on mapping plausible connections and identifying spatial flows that may reflect underlying ecological or anthropogenic processes.

#### 2.3.1. Statistical Analysis of Dispersion Plausibility

We applied statistical tests to the retained set of epidemiologically plausible connections to evaluate potential associations between dispersion plausibility and a set of categorical variables: host functional group, vaccination status, and geographic scope. The main objective of this stage was not inferential generalisation but rather the identification of internal structural trends that could inform the interpretation of vaccine-related CDV circulation. The outcome variable used in all tests was the level of potentiality, previously defined as an ordinal score ranging from 1 (highest plausibility) to 7 (lowest), based on host functional group compatibility, ecological coherence, directionality, and spatial context. We used Kruskal–Wallis H-tests to examine whether the level of potentiality differed significantly across host functional groups, evaluated separately as origin and destination. This was performed for both genotypes. We employed Mann–Whitney U tests to assess binary contrasts, specifically (i) whether the presence of at least one vaccinated host in the connection was associated with different levels of potentiality and (ii) whether connections spanning different continents showed higher, similar, or lower dispersion plausibility compared to intracontinental ones. All tests were performed independently for the America-1 and Rockborn-like genotype datasets using Jamovi v2.3.21. Variables were handled as ordinal or binary, and no assumptions of normality were made. We integrated the outcomes of these tests into the Results section and used them to support the construction of spatial representations and the refinement of the functional hypotheses.

#### 2.3.2. Spatial Processing and Vector Generation

To explore the spatial patterns underlying the network of epidemiologically plausible CDV connections, we conducted two complementary spatial analyses in ArcGIS 10.5 using vector-based methods. These analyses aimed to assess both the directional structure and the functional dispersion centres of the routes, distinguishing between the America-1 and Rockborn-like genotypes. These spatial analyses were not intended to reconstruct actual transmission chains but to identify patterns of functional coherence among plausible routes of virus spread. By stratifying directional vectors (LDMs\) and spatial centroids (MC) by host group, vaccination status, and continent, we aimed to visualise consistent structural tendencies in the geographical organisation of CDV genotypes. This approach provides a complementary interpretative layer that integrates ecological, temporal, and spatial dimensions, enhancing our understanding of virus circulation potential. First, we applied the Linear Directional Mean (LDM) tool to the connection vectors. This method calculates the average direction (azimuth) of a set of lines, as well as the angular dispersion (circular variance) and average length. The resulting output is a directional vector representing the dominant orientation of the connections. The analysis was initially performed at the global level for each genotype and subsequently repeated using the following variables as grouping factors: (a) Vaccination status of the origin and destination hosts, (b) Host functional group of the origin and destination hosts, (c) Potentiality level of the connection (ordinal scale), and (d) Continent of origin and destination. These stratified LDM analyses enabled the comparison of directional consistency across host profiles and contextual variables. We projected all line datasets in geographic coordinates (WGS84), and the analysis focused on relative directional tendencies rather than metric precision.

To complement the directional analysis with a spatial concentration perspective, we conducted a second set of analyses using the Mean Center (MC) tool. Instead of using the connection endpoints, this approach focused on the midpoints of each vector, representing the overall spatial trajectory of each plausible route. For each midpoint, a weighted mean centre was calculated using the ArcGIS Spatial Statistics tool, assigning a weight (8—plausibility level) in order to prioritise the most epidemiologically plausible connections. Separate mean centres were computed for each host category using a case field. This offered greater influence on the most epidemiologically plausible connections. Then, we calculated the mean centres for subsets of connections grouped by (i) Vaccination status (origin), (ii) Host functional group (origin), and (iii) Continent of origin. This method allowed for the identification of geographic centres of epidemiological gravity for each group, offering a complementary perspective to the linear directional means. While the LDM captured the structural flow of the connections, the MC analysis provided a spatial anchor that reflects where plausible transmission pathways tend to converge. To facilitate understanding of this spatial analysis, it is important to clarify the aim of this approach. The weighted mean centre does not represent a point of origin or a specific transmission focus but rather a spatial average that helps visualise the overall position of the connections under study. It functions as a conceptual “centre of gravity” that shifts depending on the geographic distribution of cases and the strength (plausibility) of their connections. By connecting each midpoint to its corresponding centre, the figure helps to illustrate how plausible transmission routes tend to cluster geographically, even when the actual distances between cases are large or scattered across regions.

## 3. Results

### 3.1. Reported Cases of Post-Vaccination CD or Detection of CDV Vaccine Strains in Domestic and Wild Carnivores

The scientific literature includes abundant cases of post-vaccination CD published over many decades. Among the reported studies, some authors have not attributed the case of CD to a specific vaccine strain, nor have they isolated and sequenced CDV, indicating only that it is a case associated with the inoculation of MLV-vaccine; therefore, in these cases, we cannot know to which genotype the implicated strain belongs. Other researchers have attributed CD to a specific vaccine strain, which allows it to be phylogenetically classified into Rockborn-like or America-1 genotypes. Finally, some authors have sequenced the strain of CDV and classified it into a genotype. To accurately determine whether CD was caused by post-vaccination inoculation or by infection with a field strain from vaccinal origin, the isolation, sequencing and phylogenetic classification of the strain involved can be consulted in Table S3 (Supplementary File S3).

In studies that did not sequence vaccine strains causing CD, some cases developed in vaccinated animals included domestic dogs [36,37] and captive wild carnivores like ferrets (*Mustela putorius*) [38]. In these cases, if the involved strain had undergone extra-animal dissemination within the ecosystem, its genotype could not be identified.

In general, live attenuated vaccine strains are classified into two types: those attenuated in cell lines of avian origin and those attenuated in cell lines of canine origin. Specifically, strains attenuated in cell lines of avian origin phylogenetically correspond to the America-1 genotype (e.g., Ondersteeport, Lederle, Snyder Hill, and Convac, among others). Of these, cases of post-vaccination CD in domestic dogs have been reported since 1956 [39] in South Africa, USA [40], Scotland [41], and Northern Ireland [17], where it is suggested that the strain described in 1980 has survived in the field for more than 40 years. More recent studies detected America-1 strains in domestic dogs in Poland [42], Thailand [43], Brazil [44], the USA [45,46], Italy [47], and Finland [48]. In addition, America-1 genotype has been detected in wild carnivores, such as red foxes (*Vulpes vulpes*), European mink (*Mustela lutreola*), European polecat (*Mustela putorius*) [42,49], raccoon (*Procyon lotor*), stripped skunk (*Mephitis mephitis*) [50,51,52], and also in other mustelid species [22,42,49,52,53] and wild felids [54].

Live vaccine strains attenuated in cell lines of canine origin correspond to Rockborn strains and strains sequenced as Rockborn-like. Since 1974, post-vaccination CD in domestic dogs has been reported in Australia [55], Switzerland [56], Scotland [57], Germany [58], Italy, Denmark and Hungary [16], Canada [59], USA [60], Japan [61], and very recently in New Zealand [62]. These findings show that even though MLV vaccines do not use this strain, it is still possible to find it in commercial formulations 35 years after its official withdrawal from the markets [16].

In addition, we found the trace of Rockborn or Rockborn-like genotypes in several wild carnivore species since 1976, when a report described CD in lesser panda (*Ailurus fulgens*) [63], whose strain was later sequenced as Rockborn-like [16,64], and in black-footed ferrets *(Mustela nigripes*) [65]. Since the 1980s, these strains have been detected producing CD in free-ranging and captive wild animals such as kinkajou (*Potos fiavus*) [66], gray fox [67], maned wolf (*Chrysocyon brachyurus*) [68,69], fennec fox (*Fennecus zerda*) [70,71], African wild dog (*Lycaon pictus*) [72,73,74], South American bush dog (*Speothos venaticus*) [75], European mink [76], American black bear (*Ursus americanus*) [77], and masked palm civet (*Paguma larvata*) [25].

### 3.2. Descriptive Epidemiology of CD Due to Strains of Vaccine Origin

**Figure 3:**
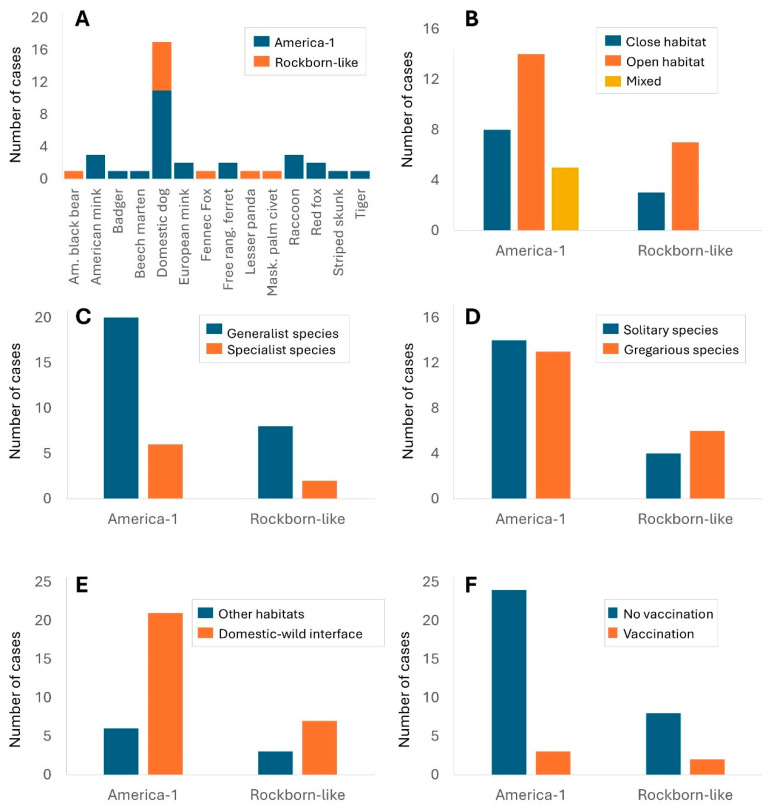
Host species and associated ecological and epidemiological characteristics for the CDV isolates analysed. (**A**) Host species identified in each case, with bars coloured by detected CDV genotype: America-1 (blue) and Rockborn-like (orange). (**B**) Habitat type used by the species, with categories coded by colour: Close habitat (blue), Open habitat (orange), and Mixed habitat (yellow). (**C**) Degree of ecological adaptability, distinguishing generalist species (blue) from specialist species (orange). (**D**) Social behaviour of the host species: solitary (blue) and gregarious (orange). (**E**) Degree of interaction with human environments (epidemiological interface with domestic animals), distinguishing cases where this interaction is absent of or minimally relevance (blue) and those involving a domestic–wild interface (orange). (**F**) Vaccination status reported for each case: no vaccination (blue) and vaccination (orange).

Cross-analysis of genotypes (America-1 and Rockborn-like) concerning species in which CDV has been isolated, and certain biological and ecological characteristics of these species, such as their presence in the human–domestic–wild interface, their environmental adaptability, their social behaviour, and their geographical distribution, reveal valuable patterns. Noteworthy findings reveal that domestic dog is both the predominant host and the species most subject to preventive vaccination measures against CDV; several species of mustelids, which are used for fur production (i.e., American mink and ferret), also stand out; red foxes and European mink are also among the main host species where vaccine-related strains cause CD. Additionally, the Rockborn-like genotype has been isolated and sequenced in uncommon hosts such as civet, fennec fox, and black bear (Figure 3A). On the other hand, isolates and sequences classified as America-1 show unexpected records in European polecat, badger (*Meles meles*) and striped skunk, highlighting a broad susceptible host range.

Analysis of biological and ecological variables reveals distinct patterns between the two large genetic groups. Specifically, the America-1 genotype predominates in species that prefer open habitats (14 species) (meadows, steppes, tundras, agroforestry ecotones, etc.). However, it can also be found in eight host species that live in closed habitats (forests, jungles) and in species that, due to their generalist behaviour, use mixed environments (five host species) (Figure 3B). In contrast, Rockborn-like has been isolated preferentially in species inhabiting open habitats (seven species), with lower representation in closed habitats (three species) and no detection in mixed environments (Figure 3B).

Regarding the environmental adaptability of species with isolated CDV from vaccine-related strains, both genotypes appear mainly in generalist species (America-1 in 21 species; Rockborn-like in 8 species), suggesting a wide potential for dissemination due to the great capacity of these species to occupy different habitats (Figure 3C). When analysing the social behaviour of species (with a focus on their gregariousness), findings reveal a prevalence almost equally distributed between solitary and gregarious species, with 13 cases recorded in gregarious species having the America-1 strain and 6 cases with Rockborn-like (Figure 3D). Finally, the presence of vaccine-related CDV isolates and sequences is particularly notable in species living in habitats at the domestic–wild interface, being high in America-1 genotypes (21 cases) and also relevant in Rockborn-like (7 cases), reinforcing the hypothesis of favoured transmission in areas of interspecific contact between wild hosts and domestic hosts (dogs) or wild hosts in captivity (Figure 3E).

**Figure 4:**
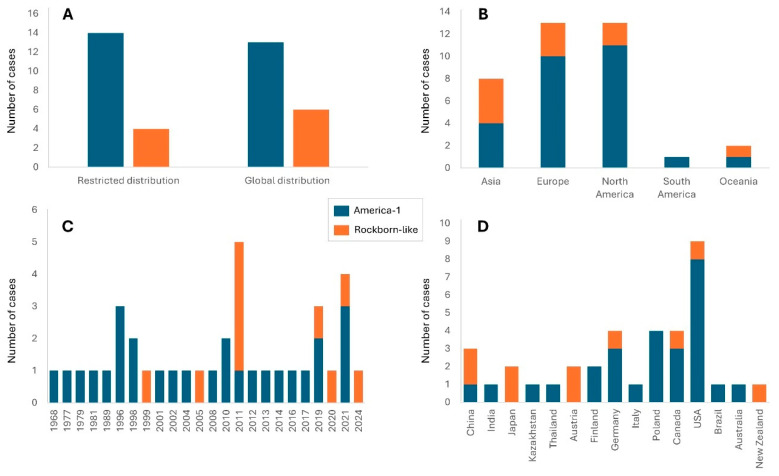
Geographical and temporal distribution of the CDV isolates analysed in this study. (**A**) Distributional range of the host species classified as either restricted or global. (**B**) Number of cases reported by continent. (**C**) Temporal distribution of records by year of publication. (**D**) Number of cases by country. Bars are coloured according to the detected CDV genotype: America-1 (blue) and Rockborn-like (orange).

Analysis of the geographical distribution by continent reveals notable differences between the two major genotypes. Specifically, isolation and sequencing of America-1 has occurred both in species with a global distribution range (13 cases) and in those with restricted geographical distribution areas (14 cases). Conversely, the species yielding the Rockborn-like genotype show a slight predominance of global distribution areas (six cases compared to four restricted) (Figure 4A). The America-1 genotype is particularly prevalent in Europe (10 records), mainly in Poland, Germany, Finland, and Italy, and North America (10 cases), both in USA and Canada. Cases have also been detected in Asia (four), Oceania (one), and South America (one). In contrast, the Rockborn-like genotype has been mainly found in Asia (four cases), followed by Europe (three), North America (two) and Oceania (one), with no cases in South America (Figure 4B,D).

The temporal distribution of sequenced vaccine-related CDV isolates shows a trajectory consistent with the evolutionary history of the virus (Figure 4C). The America-1 genotype has been detected since 1968. The number of cases of the America-1 genotype has remained stable over the decades, with peaks in 1996 and 2021. In contrast, isolates and sequences of CDV genotypes classified as Rockborn-like emerged in 1999 and show an erratic trend, with a peak in 2011 and reaching records until 2024.

### 3.3. Functional Epidemiological Profiles of Vaccine-Related Genotypes

**Figure 5:**
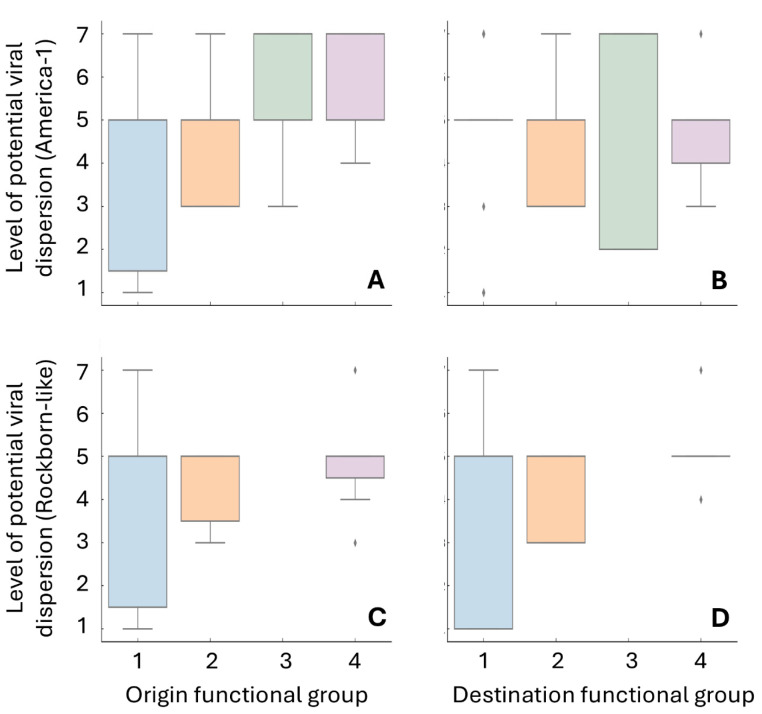
Boxplots showing the distribution of viral dispersion potential (ordinal scale: 1 = highest plausibility; 7 = lowest) across host functional groups, differentiated by genotype and host role. Panels (**A**,**B**) represent the functional group of the origin host for America-1 and the corresponding distributions for destination hosts, respectively. Similarly, panels (**C**,**D**) show these same distributions for Rockborn-like genotypes. The colour scheme is consistent across genotypes and roles: (1) domestic dog, (2) captive carnivore, (3) synanthropic mesocarnivore, and (4) wild non-synanthropic carnivores. Dots represent statistical outliers beyond 1.5 × IQR.

To characterise the ecological and epidemiological dynamics of vaccine-related CDV circulation, we conducted a comparative analysis using the curated set of documented connections involving the America-1 and Rockborn-like genotypes. The aim was to identify structural patterns in the dispersion potential of each genotype according to the functional attributes of the hosts involved. Figure 5 summarises the potential patterns of vaccine-related CDV transmission according to the functional host group, considering both America-1 and Rockborn-like genotypes and differentiating between origin and destination roles. In America-1 connections (Figure 5A,B), domestic dogs show the widest range of viral dispersion potential as origin hosts, with a median corresponding to low-potential values, suggesting frequent and plausible transmission. In contrast, synanthropic and wild non-synanthropic carnivores appear predominantly at higher potential levels, especially when acting as destinations.

Rockborn-like connections (Figure 5C,D) display a more constrained distribution, with fewer cases and reduced variability. The differences across functional groups are less pronounced, consistent with the overall lower prevalence and reduced circulation of this genotype. Captive carnivores, when implicated, appear to occupy intermediate positions within the potentiality scale.

**Figure 6:**
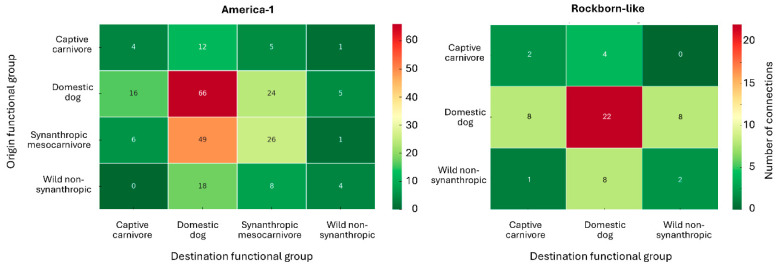
Heatmaps illustrate the number of vaccine-related CDV connections between host functional groups based on documented cases for each genotype. The matrices reflect the directionality of the transmission (origin → destination) and are colour-coded by frequency (see scale). The left panel corresponds to the America-1 genotype, and the right panel shows the Rockborn-like genotype. Domestic dogs are the main origin group in both datasets, with marked differences in transmission structure and diversity across the host functional group.

The functional profiles observed in the potentiality levels are further reinforced by the analysis of connection frequencies between origin and destination groups. To evaluate how vaccine-related transmission routes are distributed across host functional categories, we analysed the number of connections established between host functional groups for each genotype. Figure 6 shows the functional mapping of vaccine-related transmission routes, expressed as the number of connections between host functional groups for each genotype. In the America-1 dataset, domestic dogs appear as the primary source of dispersion, with a particularly strong link to other domestic dogs (*n* = 66), but also substantial connections towards synanthropic mesocarnivores (*n* = 24), captive carnivores (*n* = 16), and to a lesser extent wild non-synanthropic hosts (*n* = 5).

Synanthropic mesocarnivores are another significant source of vaccine-related genotype circulation, particularly towards domestic dogs (*n* = 49 cases) and other synanthropic host species (*n* = 26). Connections involving wild non-synanthropic hosts, although less frequent, are present across all categories.

In the case of Rockborn-like, the host functional group structure appears more constrained and less diverse. Domestic dogs dominate as both origin and destination, but with lower overall counts. Notably, connections towards wild non-synanthropic species have been rarely described, and the interface between synanthropic and captive carnivore hosts is scarcely represented.

### 3.4. Statistical Assessment of Dispersion Patterns

We assessed the potential structural differentiation of vaccine-related CDV transmission routes through non-parametric statistical tests applied to the curated dataset of documented cases. Analyses focused on identifying whether the level of potentiality, an ordinal indicator of the epidemiological plausibility of a given connection, was associated with host functional group, immunological, or geographical attributes of the hosts involved. A Kruskal–Wallis H-test (Table S4, Supplementary File S4) revealed significant differences in the level of potentiality according to the host functional group in America-1 connections, both when acting as origin (H = 18.04, *p* = 0.00043) and destination (H = 100.93, *p* < 0.001). These results support the presence of functional stratification in the transmission routes associated with this genotype. In contrast, we did not find significant differences in Rockborn-like connections (origin: H = 1.08, *p* = 0.584; destination: H = 2.27, *p* = 0.322), suggesting a less structured pattern, potentially shaped by reduced case numbers or more stochastic dynamics. We also evaluated the influence of host vaccination on transmission plausibility using the Mann–Whitney U test (Table S5, Supplementary File S4). We did not find significant differences between connections involving at least one vaccinated host and those not involving vaccinated individuals, neither for America-1 (U = 3537.5, *p* = 0.709) nor Rockborn-like (U = 376.0, *p* = 0.163).

Lastly, we conducted a comparison between intercontinental and intracontinental connections to assess whether geographical distance may be associated with reduced plausibility (Table S6, Supplementary File S4). In America-1, we did not observe a significant difference (U = 8274.0, *p* = 0.124). However, in Rockborn-like, intercontinental connections exhibited significantly higher potentiality levels (U = 612.0, *p* < 0.001), which may reflect residual traces of past Rockborn-like genotype circulation or sporadic long-distance transmission events related to the historical use of this vaccine strain.

### 3.5. Spatial Patterns of Plausible Viral Connections: Directional Structure and Functional Centres

The spatial analysis of epidemiologically plausible viral connections was deepened through two complementary approaches: the calculation of Linear Directional Means (LDM) and the identification of weighted Mean Centres (MC) based on midpoint coordinates. We performed these analyses separately for the America-1 and Rockborn-like genotypes and stratified by relevant epidemiological and contextual variables.

#### 3.5.1. Spatial Structure of Plausible Connections: Comparative Analysis by Genotype

**Figure 7:**
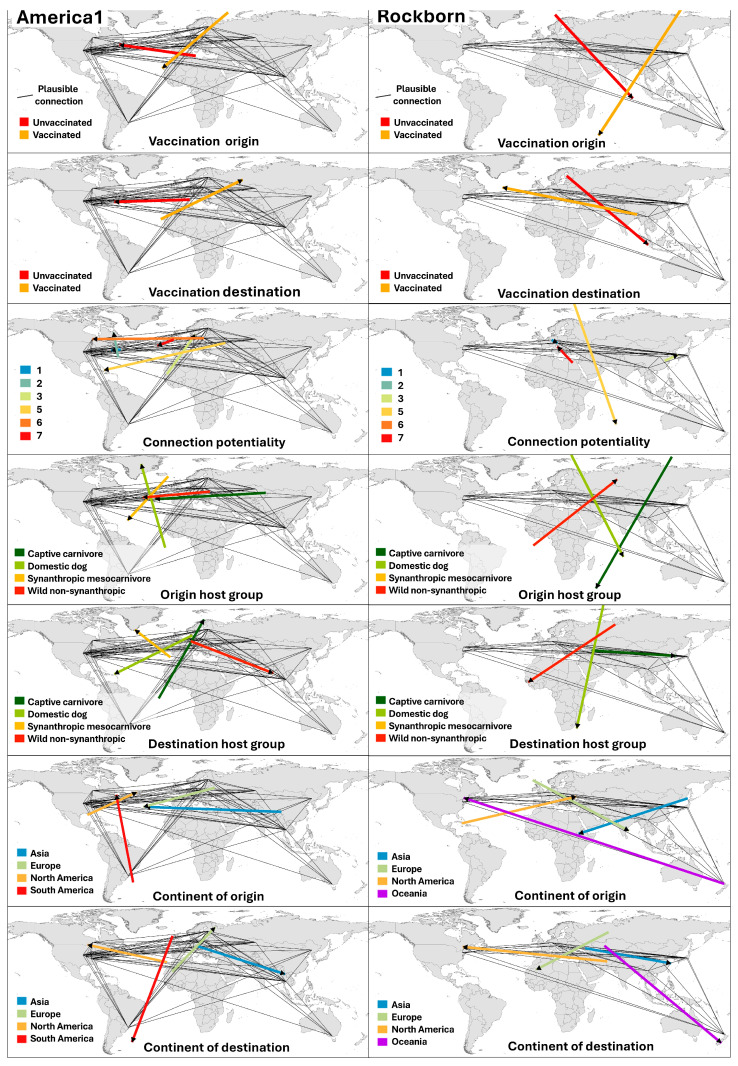
Spatial representation of directional vectors calculated for epidemiologically plausible CDV connections. The left column corresponds to the America-1 genotype and the right column to the Rockborn-like genotype. Each map shows all plausible connection lines in grey and overlaid directional vectors coloured by category. Rows represent stratification by (1) vaccination status of the origin host, (2) vaccination status of the destination host, (3) connection potentiality level (1 = most plausible, 7 = less plausible), (4) origin host functional group, (5) destination host functional group, (6) continent of origin, and (7) continent of destination. Each directional vector was generated using case fields corresponding to the categories indicated, representing the average azimuth and length of the subset of connection lines. Angular dispersion is not visualised but was calculated internally for each case.

Figure 7 shows the results of LDM of plausible CDV connections stratified by categorical variables analysis, including seven variables: vaccination status of the origin and destination hosts, connection potentiality level (1 to 7), origin and destination of host functional group, and origin and destination continents. For each category, we generated a directional vector representing the dominant orientation of the plausible routes.

(i) Vaccination status of the origin host

For America-1, the vast majority of plausible connections originated from unvaccinated hosts (235 routes), producing a consistent southeast–northwest vector, with most plausible routes beginning in the Eastern Mediterranean and reaching domestic dogs in North America. Vaccinated origin hosts (10 routes) showed a distinct trajectory, oriented southwest–northeast, mostly linked to cases in Western Europe. Although much less frequent, these vaccinated–origin connections suggest residual transmission potential or circulation among partially immunised populations. In Rockborn-like, the pattern was similar in proportion (51 unvaccinated vs. 4 vaccinated), but the directional flow was more variable. Most connections from unvaccinated origins pointed eastward from the Eastern Mediterranean toward Central and East Asia. Vaccinated origin connections, influenced by a few cases in Japan and China, showed broader dispersion and lacked a clear geographic convergence.

(ii) Vaccination status of the destination host

In America-1, unvaccinated hosts were the destination in 215 connections, producing a dominant northeast–southwest directional vector that connects the Eastern Mediterranean with North America. The cluster of these routes reinforces the concentration of plausible transmissions between Europe and the United States. Vaccinated destinations (30 connections) generated a southwest–northeast vector, originating from South America and Western Africa and directed towards Europe. This configuration reflects plausible return routes or transcontinental links between vaccinated host populations. In Rockborn-like, unvaccinated destinations (40 connections) yielded a southeastward vector from Europe and the Eastern Mediterranean to Oceania. In contrast, vaccinated destination cases (15 connections) generated a vector towards the west (orange arrow), spreading from East Asia to North America. These trajectories correspond to routes involving Asian hosts and geographically dispersed vaccine-related records.

(iii) Connection potentiality level

In America-1, the distribution of plausibility levels was broad, but with a concentration in the mid-to-high range: 28 connections were classified as Level 1, 17 as Level 2, 22 as Level 3, and 106 as Level 5. Levels 4, 6, and 7 accounted for smaller proportions. The directional vector for Level 1 (blue) was short and located over northeastern North America, with a gentle northeast–southwest inclination. Level 2 vector (green-blue) also remained in North America but displayed a near south–north orientation. In contrast, Level 3 vector (light green) represented a transcontinental route from West Africa to Europe, with a clear southwest–northeast direction. These short vector lengths in the highest plausibility levels reflect the local concentration and spatial consistency of the connections, indicating minimal geographic dispersion. In contrast, connections with lower plausibility levels, particularly Level 5 (106 connections) and Level 7 (66 connections), were spatially broader and directionally more variable. Vectors associated with these levels tended to be longer, often linking regions such as South America, West Africa, and North America, reflecting the greater spatial spread and lower cohesion of these hypothetical pathways. In Rockborn-like, the plausibility levels present were 1, 3, 4, 5, and 7, with the majority of cases concentrated in Level 5 (36 connections). Level 1 vector (blue, 10 connections) originated in Europe and followed a southeastward trajectory towards cases in Oceania (New Zealand). This vector was relatively short, indicating a more localised structure among the most plausible routes. Level 3 vector (light green, five connections) linked Central Asia to East Asia, forming an eastward flow with moderate extension. In contrast, Level 5 vectors, though more numerous, produced longer and less cohesive trajectories, including transoceanic connections such as those from East Asia toward Oceania. The two Level 7 connections showed no directional consistency and were spatially scattered. The variability in vector lengths, especially in the intermediate and lower plausibility levels, highlights the contrast between localised, high-confidence dispersal patterns and the more exploratory nature of long-distance, lower-confidence scenarios.

(iv) Host functional group of origin

In America-1, domestic dogs accounted for 111 plausible connections, generating a long south-to-north vector, strongly influenced by South American cases. The geographical location of origin points between South America and Europe reflects a bidirectional dispersion, although with greater density towards North America. Synanthropic mesocarnivores (82 connections) produced a similarly dispersed vector, oriented predominantly east to west, suggesting the influence of European hosts on American destinations.

Wild non-synanthropic carnivores (30 connections) showed a defined east-to-west directional vector, with origins concentrated in Europe and plausible destinations in North America. Similarly, captive carnivores (22 connections) exhibited a long westward vector but origin points predominantly in Asia, likely influenced by clustered records in China, and connections extending toward Europe and North America. The variation in vector length and orientation across groups reflects both ecological differentiation and the broad spatial distribution of origin hosts. Longer vectors, particularly for wild and captive carnivores, indicate greater geographical spread or lower clustering of source cases.

In Rockborn-like, domestic dogs again dominated the origin category (38 of 55 connections), producing a long vector with north–northeast to south–southwest directionality, linking European origins with destinations in Asia and Oceania. Wild non-synanthropic carnivores (11 connections) produced a vector of similar length but opposite orientation (southwest to northeast), reflecting a dispersed and structurally irregular spatial distribution. Captive carnivores (six connections), though limited in number, also displayed a fragmented directional flow with a north-to-southeast trajectory, likely driven by sparse Asian origin points. Compared to America-1, Rockborn-like vectors involved fewer host groups and showed a more diffuse spatial configuration, likely influenced by the smaller sample size and the geographic concentration of records in Asia and Europe.

(v) Host functional group of destination

In America-1, domestic dogs were the destination in 145 plausible connections, forming a dominant northeast–southwest vector between Europe and America. This reflects the high density of transatlantic connections involving unvaccinated dogs in both regions. Synanthropic mesocarnivores (63 connections) showed a westward vector, caused in this case by the influence of Asian and North American CDV-positive records, with moderate spatial dispersion. Captive carnivores (26 connections) generated a southwest–northeast vector connecting cases in South America with those in Europe. Meanwhile, wild non-synanthropic carnivores (11 connections) showed a vector pattern oriented towards Asia, mainly from Europe, but also influenced by cases in North America. The variety of directional vectors reflects the functional role of the host species and the heterogeneity of the destination contexts in America-1. The longest vectors are particularly evident between captive and synanthropic destinations, indicating a lower specific local weight. In Rockborn-like, the three host functional groups of destination, domestic dogs, captive carnivores, and wild non-synanthropic carnivores showed marked heterogeneity in their directional means. Domestic dogs, representing the majority of connections (34 of 55), displayed a long north–south vector, indicating a high geographic dispersion of destination cases and the absence of a dominant spatial trend. Captive carnivores (11 connections) showed an eastward vector, driven by the influence of Asian cases with plausible origins in Europe. Wild non-synanthropic carnivores (10 connections) produced an intermediate directional pattern, shaped by transatlantic connections from Europe to North America. Overall, the spatial orientation of Rockborn-like destination hosts was less cohesive than in America-1, reflecting the broader geographical dispersion and uneven sampling across host categories.

(vi) Continent of origin

In America-1, the most plausible connections originated in Europe (107) and North America (96), forming a well-defined bidirectional corridor over the North Atlantic. These origins produced clearly aligned vectors: the European origin vector pointed toward North America and vice versa. This pattern reflects the high plausibility of reciprocal connections between regions with numerous cases, primarily involving domestic dogs. Asia contributed 34 connections, generating a clearly westward vector directed toward both Europe and, more strongly, North America. South America (eight connections) produced a northward vector, indicating plausible routes mostly confined within the Americas. In summary, the combination of European and North American origins defined the dominant spatial structure in the LDM analysis, while other continents contributed to secondary or peripheral flows.

In Rockborn-like, the directional pattern was more defined, likely due to the smaller number of records compared to America-1. European origins (26) produced an eastward vector toward Asia and Oceania, while Asian origins (21) generated a northwestward vector directed toward North America, with additional connections to Europe, indicating greater dispersion. North America (six) and Oceania (two) contributed marginally, but their vectors were spatially consistent: the North American vector pointed clearly toward Europe, while the Oceanian vector followed a northeastward trajectory toward North America. Compared to America-1, Rockborn-like origins were more fragmented and polarised, generating multidirectional flows with limited overlap between continents.

(vii) Continent of destination

In America-1, plausible CDV destinations were concentrated in North America (113 connections) and Europe (89), followed by Asia (42) and South America (12). In Rockborn-like, Asia (24) and Europe (23) accounted for most destinations, while North America (8) and Oceania (7) had more limited representation. Notably, across both genotypes, the linear directional means of destination vectors displayed mirror-like patterns with respect to the origin vectors described in the previous section. This symmetry reflects a strong internal coherence in the plausibility structure of the network: the spatial direction of origin-to-destination flows is reversed in the destination-based vectors, as expected from the bidirectional nature of the dataset. This consistency supports the robustness of the spatial structuring observed and highlights the complementarity between origin and destination perspectives in the functional interpretation of potential CDV transmission routes.

#### 3.5.2. Spatial Aggregation of Plausible Pathways by Host and Epidemiological Attributes

**Figure 8:**
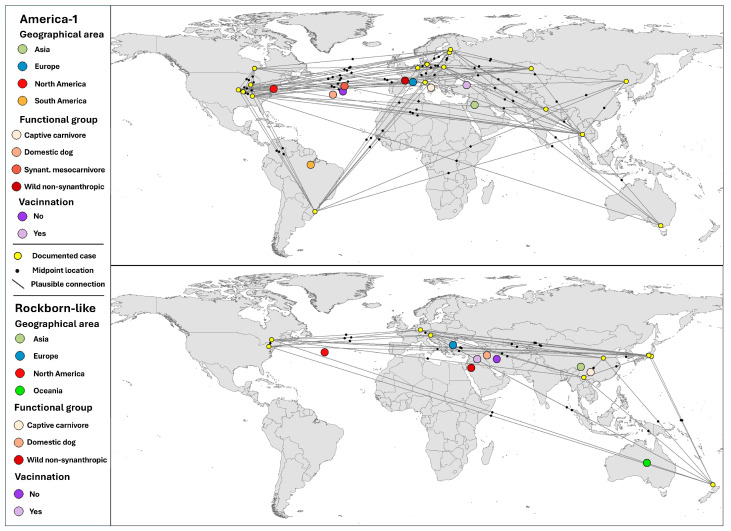
Weighted mean centres of connection midpoints stratified by origin host attributes. Spatial distribution of mean centres calculated from the midpoints of each plausible CDV connection vector, weighted by the inverse of the potentiality level (weight = 8 − level) to prioritise the most plausible connections. The upper panel corresponds to the America-1 genotype and the lower panel to the Rockborn-like genotype. Points are grouped and symbolised according to three categorical variables associated with the origin host: geographical area (continent), host functional group (domestic dog, synanthropic mesocarnivore, captive carnivore, wild non-synanthropic carnivore), and vaccination status (0 = unvaccinated, 1 = vaccinated). Each coloured symbol represents the weighted spatial centre of the midpoints for all plausible connections in that category. Yellow dots represent documented CDV cases retrieved from the literature, which serve as the basis for generating plausible transmission routes, shown as grey connecting lines. Black dots correspond to the individual midpoints of those routes, and coloured centroids reflect the aggregated mean centre for each host category.

Figure 8 displays the spatial distribution of case records by genotype using a weighted mean centre approach to illustrate the overall directional trend in virus reporting. For each genotype, individual records are georeferenced and connected to their corresponding central point, calculated as the geographic centroid of the distribution. These centroids do not represent transmission foci but rather summarise the spatial structure of the plausible connections, indicating the geographical balance of the recorded cases. They should be interpreted as conceptual anchors, not as actual points of viral emergence.

In America-1, the spatial distribution of centres reveals distinct geographic patterns depending on the stratifying variable.

(i) Geographical area: Mean centres for cases originating in Europe and North America appear clearly separated, reflecting two dense and internally consistent nuclei (Figure 8, points of origin marked in yellow). In contrast, the midpoints associated with South America and Asia were located further away from the original locations of these continents, revealing a greater weight among the connections of the cases found in North America and Europe, respectively, which does not indicate a source of transmission in these locations but rather the balance between the influences of geographically distributed cases.

(ii) Host functional group: Domestic dogs (second level of red tone) concentrate towards the western Atlantic, influenced by multiple plausible connections between European and North American dogs. Similarly, synanthropic mesocarnivores (third level of red), located in an intermediate position between these same continents, reflect a significant weight of their cases with a broader ecological range and more dispersed centres. Meanwhile, wild non-synanthropic carnivores (dark red) and captive species (pale red) have isolated or peripheral centroids, which is consistent with their limited geographical spread.

(iii) Vaccination status: Unvaccinated origin hosts (purple) show a concentration similar to the dog cluster, while vaccinated origins (light violet) shift eastwards, in line with the European dominance among vaccinated individuals. The apparent central position of some centroids over the Atlantic (e.g., vaccination status 0) results from plausible transatlantic connections and should be interpreted as a conceptual midpoint, not as a real epicentre of viral movement.

In Rockborn-like, the spatial structure of the midpoints is less cohesive, consistent with the lower number of records and the more fragmented nature of the plausible connections. Nevertheless, some grouping trends can be distinguished when stratified by host variables.

(i) Geographic area: The centres represent Asia, Europe, North America, and Oceania, but not South America. Compared to America-1, the continental centres of the Rockborn-like group are generally more dispersed, but with a clear representation of Asian cases. It should be noted that the green marker representing oceanic cases appears in Australia, reflecting possible connections involving hosts from other geographical environments with those found in the scientific literature in New Zealand. The centre associated with North America (red) located in the Atlantic near the east coast, like the European centroid (blue), is slightly shifted to the southeast with respect to the equivalent point in America-1, indicating that the location of the central weight of the other continents is tilted towards Asia.

(ii) Host functional group: Domestic dogs (second level of red tone) again account for most connections, but the resulting centre is not located over their most frequent origin points but rather near the Eastern Mediterranean. This shift is explained by the influence of plausible east–west connections, especially those involving cases from East Asia (notably China and Japan), which draw the centroid away from Europe and result in an intermediate geographic position. Wild non-synanthropic carnivores (dark red) also show a centroid over the Middle East, similarly influenced by transcontinental connections. Captive carnivores (pale red) present a more localised distribution centred over East Asia, where most of their records originate.

(iii) Vaccination status: Unvaccinated origin hosts (purple) display a spatial centre located over the Middle East, reflecting a balance between cases from Europe and Asia. This pattern is not due to the presence of cases in the region itself but to the geographic pull of plausible connections spanning long distances. Vaccinated hosts (light violet), more frequently associated with Asian records, shift the centroid further east, towards East Asia. Compared to America-1, where vaccination status produced a well-defined divergence across the Atlantic, the Rockborn-like centroids appear more condensed and regionally displaced due to the smaller sample size and the strong influence of cases from eastern Asia.

## 4. Discussion

*Morbillivirus canis* (CDV) has been described in families of wild carnivores such as Canidae, Mustelidae, Procyonidae, Mephitidae, Phocidae, Ursidae, Felidae, Hyaenidae, and Viverridae [11,78,79,80]. In addition, it has been described in aquatic mammals [81], non-human primates belonging to Cercopithecidae and other families [82,83,84], other mammals belonging to Myrmecophagidae [85], Hystricidae and Sciuridae families [47], Artiodactyla [86], and Proboscidea orders [12,87]. We can therefore confirm the high degree of dispersion of CDV and, on the other hand, the large network of primary and secondary hosts in which this virus can be detected.

Tracking the spread of multi-host pathogens such as CDV, especially in the context of vaccine-related strains [10], presents significant challenges due to the complexity of host–pathogen interactions and the influence of the environment [28,31,88,89]. Our study, based on a scoping review and exploratory analysis of documented cases of CDV, sought to elucidate the spatial and host functional patterns associated with the transmission of the America-1 and Rockborn-like genotypes. The results obtained, although derived from presence/absence data and not from confirmed transmission chains, provide a host functional group framework for interpreting how host characteristics and geographical context influence the potential spread of these strains. The spatial analysis of plausible CDV connections, based on directional means and midpoint-derived mean centres, offers a structured representation of the virus’s potential dispersal pathways, grounded in host functional group rather than direct transmission evidence. While the data do not reflect confirmed transmission events, they emerge from a review of the literature and a rigorous plausibility framework, allowing for hypothesis-driven exploration of spatial patterns.

Regarding the specific biological characteristics and degree of ecological plasticity of the host species, the America-1 and Rockborn-like genotypes were predominantly detected in species with generalist habits, suggesting a broad dissemination potential due to their ability to adapt to and effectively occupy diverse habitats (open, closed, and mixed). The notable presence of these genotypes in species inhabiting domestic–wildlife interface areas strongly supports the hypothesis that transmission is favoured where intra- and interspecies contacts occur. Furthermore, records are distributed among solitary and gregarious species, with a prevalence of gregarious species in cases of CD caused by documented vaccine strains [90]. This could imply that social behaviour influences contact rates and therefore transmission within and between populations. In this regard, phylodynamic studies have identified recombination events of vaccine strains isolated in domestic dogs (from MLV vaccines, Lederle, and Convac strains) with strains from raccoons [34]. The detection of this type of phenomenon suggests interspecies contact between these species at a shared habitat interface. These results underscore the importance of considering both host diversity and the phylogenetic origin of viruses that cause CD in assessing the epidemiological impact of the virus.

The combination of direct contact transmission and the generalist, gregarious habits of species that inhabit ecotones becomes risk factors for the presence of CDV in synanthropic mesocarnivores [32]. Not all species inhabiting ecotones have the same susceptibility to infection, nor do they have the same capacity to spread the infection to other species [28]. An increasing gradient of sensitivity to CDV has been demonstrated from red foxes, raccoons, and skunks to grey foxes, with the latter species being the most sensitive to infection and, due to its specific habitat selection, the species most likely to cause spill-back among other species in an ecotone [28,91]. So, understanding the ecology of interspecific social relationships between domestic and wild mesocarnivores in shared ecotones can help identify differential risk factors in species and spaces.

One of the main conclusions is the functional and spatial differentiation of plausible transmission routes according to vaccine genotype and host functional group. The Onderstepoort strain, which is the prototype of the America-1 lineage, was developed in 1939 from a CDV isolate from foxes raised on farms in North America; the strain was originally transmitted in series in ferrets but was further attenuated by serial passages in embryonated chicken eggs [92]. Since the 1950s, this egg-attenuated strain has dominated the market and is present in most vaccines available today [16,17].

For the America-1 genotype, domestic dogs emerge as the main source of dispersal and the host functional group with the widest range of viral dispersal potential, with strong connections to other domestic dogs, synanthropic mesocarnivores, captive carnivores and, to a lesser extent, non-synanthropic wild hosts. This highlights their central epidemiological role as a reservoir and primary host spreader of vaccine-related strains, particularly in the case of America-1 (where the host functional group epidemiology appears more polarised between host species functional groups), serving as a bridge between different ecological landscapes (urban, peri-urban, natural). The analysis of Linear Mean Directions (LDMs) reinforces this centrality, showing that connections originating or ending in domestic dogs have predominant and more stable directions (North–South or East–West) in the case of America-1. Similarly, the centres of gravity (Mean Centres, MCs) for domestic dogs are concentrated in key regions such as the North Atlantic and Western Europe, consistent with the high density of domestic dogs and human activities in these areas. Anis et al. [46] suggest that the America-1 strain found in a domestic dog in the USA has a wild origin and that this clade continues to circulate in the territory; the most likely explanation for this particular animal is that it was exposed to the America-1 strain from wild animals [46]. However, given this finding, it suggests that the America-1 clade continues to circulate in wildlife in the USA, even though current vaccines effectively keep these strains out of the domestic dog population suggesting global spread associated with vaccine circulation. This distribution pattern probably reflects the historical influence of domestic dog vaccination campaigns in these regions.

Synanthropic mesocarnivores (such as foxes, raccoons, skunks, and badgers) also play a significant role as a source of vaccine-related CDV circulation, particularly to domestic dogs and other synanthropic hosts. Their adaptability to peri-urban habitats (i.e., domestic–wildlife interface) and their increased densities and contact rates [93] in anthropised areas make them important viral spreaders in host scenarios [29,94]. While domestic dogs tend to show more stable directions, synanthropic mesocarnivores may exhibit more diffuse directions (Northeast–Southwest), which could reflect their movements in these complex ecotones. In highly anthropised areas, including the ecotones that form the human–domestic–wild interface, abundant trophic resources and refuge for wild mesocarnivores with generalist behaviour are offered [95]. This phenomenon frequently results in elevated population densities for these species and subsequently a rise in both intra- and interspecific contact rates between wild and domestic hosts, particularly domestic dogs. Therefore, the direct consequence is an amplified risk of transmission and spread of pathogens such as CDV [14,90]. Within these epidemiological contexts, pathogen exchange can occur bidirectionally: from domestic dogs to synanthropic mesocarnivores and from the latter back to domestic dogs [13]. In our study, connections involving wild non-synanthropic carnivore species, although less frequent, are present across all categories, suggesting secondary routes or spillover events, mainly involving domestic dogs and synanthropic mesocarnivores. This reinforces the hypothesis that periurban areas are potential zones of host interactions and, consequently, of viral persistence and intra- and interspecific transmission.

Captive carnivores, when involved, show highly variable connection directions and more peripheral or dispersed centres of gravity. This is consistent with their greater dependence on human transport (zoos, rescue centres, breeding centres, etc.), which can generate long-range or unusual dispersal routes from a purely ecological perspective. It is very curious to note that isolates of strains from the America-1 lineage (AY445077/98-2645; AY445077/98-2646; AY445077/98-2654) in raccoons living around a zoo in USA [50] were non-artificial vaccine strains [96], which have undergone recombination from two ancestors whose hosts were a mink from China isolated in an unknown year (EU726268/CDV3) and a domestic dog from USA, isolated in 2004 (EU716337/164071) [97].

Ke et al. [34] suggest that a significant driver of CDV recombination is the capacity of vaccine strains (both America-1 and Rockborn-like genotypes) to adapt to new host species. This adaptation subsequently facilitates their spread within the ecosystem and leads to their recombination with wild-type strains. In the present study, the vaccination status of the source or destination host did not show a statistically significant association with the level of potentiality of the documented connections. However, the LDM spatial analysis did suggest that connections related to vaccinated animals may show more spatially variable or angled trajectories (North-South or Southeast in America-1, opposite directions in Rockborn-like) compared to unvaccinated animals. This could mean that dispersal from or to vaccinated animals (potentially related to shedding or residual/reversion virulence) is less geographically predictable and may be more linked to specific events or human mobility.

The Rockborn-like genotype showed a more restricted and less structured host functional group and spatial dispersal pattern than America-1. Although domestic dogs are also the key species in virus transmission as the source and destination for this genotype, the overall number of described cases is lower compared to the America-1 strain, and the diversity of host functional group connections is reduced. In fact, scarce studies documented the transmission of Rockborn-like strains to wild non-synanthropic species and represented the connection between synanthropic and captive carnivore hosts. The lower prevalence of Rockborn-like, as well as its restricted transmission pattern in the analysed data and the more erratic spatial patterns (greater angular dispersion in LDM and more fragmented MC centres), are consistent with its historical withdrawal from commercial vaccines the in the 1990s and their reduced global circulation observed in recent decades [16]. However, the persistence of Rockborn-like strain cases in Asia, Europe, and Oceania, and also the association of intercontinental connections with significantly higher (less plausible) levels of potentiality suggest that current records of these cases are residual traces of circulation or sporadic long-distance transmission events, possibly related to ancient spreading phenomena or to ecological and epidemiological factors that favour the persistence of Rockborn-like strains in unvaccinated carnivore populations.

Moreover, there are still some concerns that Rockborn strains might be in commercial vaccines [98]. The recent detection of Rockborn-like in New Zealand [62] and Canada [59], years after the commercial withdrawal of these vaccine types, suggest that these vaccine strains might still be present in authorised vaccine formulations or perhaps have adapted to carnivore species community occupying the ecosystems where these strains were detected, becoming capable of spread. In this regard, canine-derived CDV vaccines pose a risk. Indeed, vaccine-related CD has been frequently described in non-domestic species following vaccination with Rockborn strains, including African wild dog [74], lesser panda [63,64] and recently fennec fox [71]. The spatial pattern exhibited by the Rockborn-like genotype supports the idea that, even in a context of withdrawal from commercial circulation, due to frequent virulence reversion after vaccination, this strain may have found favourable ecological and environmental conditions for its persistence in unvaccinated domestic and wild hosts, leading to its diversification in Asian and European regions. Therefore, we observed spatial dispersion, requiring specific surveillance, and consequently any vaccine containing a strain similar to Rockborn should be avoided in wild species.

From the evidence that CDV MLV vaccines can induce symptomatic disease and even mortality in certain susceptible species, as they retain their ability to replicate in vaccinated animals [14,26], we are currently at a crossroads regarding the preventive use of MLV vaccines. Although these MLV vaccines produce the best humoral and cellular immunity [15], this benefit is frequently overshadowed by their potential to cause disease symptoms, host death, and vaccine strains sharing phenomena. The implications of our findings are significant. The circulation of CDV vaccine strains (particularly the America-1 strains and, to a lesser extent, Rockborn-like) and their adaptations to new host species and new ecological and epidemiological factors pose a threat to susceptible wild carnivore populations. Spillover from domestic dogs and synanthropic mesocarnivores to wild species could result in outbreaks with high mortality rates [99]. The continued detection of vaccine strains in a wide range of host species, including some rare or threatened species, highlights the need to consider the potential risk associated with the use of MLV vaccines, which, although effective in inducing immunity, can cause disease and be shed into the environment by certain susceptible species. This raises the controversy surrounding the vaccination of wild species at risk.

Recombination with wild strains [97] and genetic drift may contribute to the evolution of vaccine strains once they circulate outside the context of controlled vaccination. This highlights the importance of developing vaccine strategies that minimise viral shedding phenomena and residual virulence, seeking safe and effective vaccines for wild susceptible species. There is a need to find vaccine control strategies based on vaccines that produce adequate humoral and cellular immunity and long-lasting immunity, but which are not capable of reproducing the disease in vaccinated animals, thereby preventing the spread of vaccine strains and their possible transmission to other susceptible species.

It is crucial to recognise the limitations of our approach. The analysis is based strictly on data on the presence/absence of vaccine genotypes documented in the scientific literature. We cannot infer direct chains of transmission or perform formal phylogenetic, phylodynamic, or phylogeographic analyses. The patterns observed are influenced by biases inherent in global surveillance, diagnostic capacity, and scientific publication intensity, with an overrepresentation of regions such as Europe and North America. Therefore, the plausible network of connections should be considered a functional proxy representing where and in whom these strains could have circulated under the observed conditions, rather than an empirical map of transmission.

Despite these limitations, the results of the LDM and MC spatial analysis reveal a consistent and non-random structure in the plausible connections, which aligns with our conceptual model of functional dispersal through urban, peri-urban, and wild interfaces. The observed spatial and functional differentiation highlights how host ecology, vaccination dynamics, and geographical context interact to shape the potential spread of vaccine-related CDV strains. Our statistical analysis supports the presence of structured dispersion patterns in America-1 connections, where the host functional group appears to play a key role. In contrast, the patterns observed for Rockborn-like suggest a more limited and less differentiated set of transmission scenarios. While vaccination status was not statistically associated with transmission plausibility, the clear difference found in the geographical comparison between America-1 and Rockborn-like reinforces the hypothesis of occasional long-distance spread or the latter or persistent circulation following vaccine withdrawal. These results highlight the importance of considering both ecological and spatial factors when interpreting the dynamics of vaccine-related CDV transmission.

In conclusion, despite the limitations inherent in the literature-derived data, our study provides a novel functional and spatial perspective on the potential spread of vaccine-related CDV strains. It highlights the key epidemiological role of domestic dogs and synanthropic mesocarnivores, the influence of host ecological and social behaviours, and the relevance of domestic–wild interfaces as hotspots for interaction. The distinct patterns observed for America-1 and Rockborn-like strains reflect their different vaccination histories. A major limitation of this study lies in the relatively small number of available CDV records, particularly for Rockborn-like and certain host categories, which restricts the statistical robustness of some spatial metrics. However, the use of plausibility-based filtering and stratified spatial approaches mitigates these constraints and enables the identification of consistent directional and geographical trends. These findings should be interpreted as exploratory and hypothesis-generating, complementing conventional phylogenetic and epidemiological approaches. Visualising spatially plausible connections may be useful for prioritising surveillance areas and refining hypotheses about the ecology and epidemiology of CDV in complex host landscapes, guiding future research that integrates phylogeographic analyses based on genomic sequences and more systematic sampling.

## 5. Conclusions

Key epidemiological role of domestic dogs and synanthropic mesocarnivores: Domestic dogs emerge as the primary source of dispersal and the host functional group with the widest range of viral dispersal potential for the America-1 genotype, showing strong connections to other domestic dogs, synanthropic mesocarnivores (such as foxes, raccoons, skunks), and captive carnivores. This underscores their central epidemiological role as a reservoir and primary host spreader of vaccine-related strains, particularly for America-1, serving as a bridge between different ecological landscapes (urban, peri-urban, natural). Synanthropic mesocarnivores also play a significant role as a source of vaccine-related CDV circulation, especially to domestic dogs and other synanthropic hosts.

Influence of host ecology, behaviour, and interface areas: CDV transmission is favoured in domestic–wildlife interface areas (ecotones) where intra- and interspecies contacts occur. America-1 and Rockborn-like genotypes are predominantly detected in species with generalist and gregarious habits, suggesting broad dissemination potential due to their adaptability to diverse habitats. Ecological factors and host social behaviours are crucial for transmission. We identified peri-urban areas as potential zones for host interactions and viral persistence. The analysis suggests that understanding the ecology of interspecific social relationships between domestic and wild mesocarnivores in shared ecotones can help identify differential risk factors in species and spaces.

Differentiation of transmission patterns between America-1 and Rockborn-like genotypes: The America-1 genotype exhibits a more structured functional and spatial dispersal pattern and a broader range of viral dispersal potential, reflecting its historical influence from global vaccination campaigns. In contrast, the Rockborn-like genotype shows a more restricted and less structured pattern, with a lower number of cases and reduced diversity of connections among host functional groups. The spatial patterns for Rockborn-like are more erratic, consistent with its reduced global circulation in recent decades.

Risk from vaccine strains and need for safe vaccines: The circulation and adaptation of CDV vaccine strains (America-1 and, to a lesser extent, Rockborn-like) pose a threat to susceptible wild carnivore populations. Spillover from domestic dogs and synanthropic mesocarnivores to wild species could lead to outbreaks with high mortality rates. The continued detection of vaccine strains in a wide range of species highlights the need to consider the risk associated with the use of modified live virus (MLV) vaccines, which, although effective in inducing immunity, can cause disease and be shed into the environment. This emphasises the importance of developing vaccination strategies that minimise viral shedding and residual virulence, seeking safe and effective vaccines for susceptible wild species.

Importance of ecological and spatial factors for surveillance: The study concludes that it is crucial to consider both ecological and spatial factors when interpreting the dynamics of vaccine-related CDV transmission. The functional and spatial differentiation observed in plausible transmission routes highlights how host ecology, vaccination dynamics, and geographical context interact to shape the potential spread of these strains. Visualising these spatially plausible connections is useful for prioritising surveillance areas and refining hypotheses about CDV ecology and epidemiology in complex host landscapes.

## Data Availability

Everything related to systematic searches in bibliographic sources can be consulted at Archive.org (https://archive.org/details/osf-registrations-kndfv-v1, accessed on 24 July 2025) or through the DOI https://doi.org/10.17605/OSF.IO/KNDFV (accessed on 24 July 2025).

## Supplementary Materials

The following supporting information can be downloaded at: https://www.mdpi.com/article/10.3390/v17081045/s1, Supplementary File S1: Table S1. PRISMA checklist [100]. Supplementary File S2: Table S2. List of the 24 articles included in this review and spatial meta-analysis. Supplementary File S3: Table S3. Reported Canine Distemper due to America-1/Rockborn-like strains. Host species, post-vaccination distemper or infection with a vaccine field strain (Post-vac CD), sequencing and genotype of the strain are shown. Supplementary File S4: Table S4. Kruskal–Wallis H-tests were applied to evaluate differences in the level of potentiality (ordinal scale from 1 to 7) according to the functional group of the host species, considered either as origin or destination of the connection. Table S5. Mann–Whitney U tests were used to compare the level of potentiality between connections involving at least one vaccinated host and those not involving vaccinated individuals. Table S6. Mann–Whitney U tests were applied to assess differences in the level of potentiality between intercontinental and intracontinental connections.
