# Supplementary material for: Tracking the Spatial and Functional Dispersion of Vaccine-Related Canine Distemper Virus Genotypes: Insights from a Global Scoping Review
Source: Viruses. 2025 Jul 27;17(8):1045. doi: 10.3390/v17081045 (PMC12390544; doi:10.3390/v17081045)
Supplement: Supplementary file 1 [file viruses-17-01045-s001.zip › SupFile 2-Table S2_List of Authors.pdf]

Supplementary Material 2.Table S2. Articles included in the scoping review and meta-analysis (*n* = 24).

ID	Year	Title	Journal	Reference
1	2025	Investigation of post-vaccinal canine distemper involving the Rockborn-like strain in nine puppies in New Zealand	New Zealand Veterinary Journal	Gulliver et al., 2025
2	2024	Post-vaccinal distemper-like disease in two dog litters with confirmed infection of vaccine virus strain	Comparative Immunology Microbiology and Infectious Diseases	Pekkarinen et al., 2024
3	2024	Canine distemper virus infection of vaccinal origin in a 14-week-old puppy	Journal of Veterinary Diagnostic Investigation	Rätsep & Ojkic, 2024
4	2022	Molecular and pathological screening of canine distemper virus in Asiatic lions, tigers, leopards, snow leopards, clouded leopards, leopard cats, jungle cats, civet cats, fishing cat, and jaguar of different states, India	Infection, Genetics and Evolution	Kadam et al., 2022
5	2022	A novel and highly divergent canine distemper virus lineage causing distemper in ferrets in Australia	Virology	George et al., 2022
6	2021	Insight into an outbreak of canine distemper virus infection in masked palm civets in China	Frontiers in Veterinary Science	Shi et al., 2021
7	2021	A canine distemper virus retrospective study conducted from 2011 to 2019 in Central Italy (Latium and Tuscany regions)	Viruses	Ricci et al., 2021
8	2021	Genetic characterization of canine distemper virus from wild and domestic animal submissions to diagnostic facilities in Canada	Preventive Veterinary Medicine	Giacinti et al., 2021
9	2020	Molecular evidence for vaccine-induced canine distemper virus and canine adenovirus 2 coinfection in a fennec fox	Journal of Veterinary Diagnostic Investigation	Tamukai et al., 2020
10	2018	Phylogenetic analysis of the wild-type strains of canine distemper virus circulating in the United States	Virology Journal	Anis et al., 2018
11	2017	Homologous recombination is a force in the evolution of canine distemper virus	PLoS ONE	Yuan et al., 2017
12	2014	Real-time reverse transcription polymerase chain reaction method for detection of Canine distemper virus modified live vaccine shedding for differentiation from infection with wild-type strains	Journal of Veterinary Diagnostic Investigation	Wilkes et al., 2014

13	2013	First report of clinical disease associated with canine distemper virus infection in a wild black bear (*Ursus americana*)	Journal of Wildlife Diseases	Cottrell et al., 2013
14	2013	Detection and differentiation of field and vaccine strains of canine distemper virus using reverse transcription followed by nested real time PCR (RT-nqPCR) and RFLP analysis	Journal of Virological Methods	Fischer et al., 2013
15	2011	Lights and shades on an historical vaccine canine distemper virus, the Rockborn strain	Vaccine	Martella et al., 2011
16	2007	Genotyping canine distemper virus (CDV) by a hemi-nested multiplex PCR provides a rapid approach for investigation of CDV outbreaks	Veterinary Microbiology	Martella et al., 2007
17	2005	Phylogenetic and restriction fragment length polymorphism analyses of hemagglutinin (H) protein of canine distemper virus isolates from domestic dogs in Japan	Virus Research	Uema et al., 2005
18	2005	Nucleotide sequence analysis of nucleocapsid protein gene of canine distemper virus isolates in Thailand	Veterinary Microbiology	Keawcharoen et al., 2005
19	2005	Phylogenetic characterization of canine distemper viruses detected in naturally infected dogs in North America	Journal of Clinical Microbiology	Pardo et al., 2005
20	2004	Genetically distant American canine distemper virus lineages have recently caused epizootics with somewhat different characteristics in raccoons living around a large suburban zoo in the USA	Virology Journal	Lednicky et al., 2004
21	2003	Canine distemper of vaccine origin in European mink, *Mustela lutreola* - a case report	Veterinary Microbiology	Ek-Kommonen et al., 2003
22	2003	Sequence analysis of the fragment of the phosphoprotein gene of Polish distemper virus isolates	Archives of Virology	Rzezutka & Mizak, 2003
23	2000	Epizootiological investigations of canine distemper virus in free-ranging carnivores from Germany	Veterinary Microbiology	Frölich et al., 2000
24	1999	Detection of canine distemper virus nucleoprotein RNA by reverse transcription-PCR using serum, whole blood, and cerebrospinal fluid from dogs with distemper	Journal of Clinical Microbiology	Frisk et al., 1999

REFERENCES

Anis, E., Newell, T. K., Dyer, N., & Wilkes, R. P. (2018). Phylogenetic analysis of the wild-type strains of canine distemper virus circulating in the United States. *Virology Journal, 15*(1). https://doi.org/10.1186/s12985-018-1027-2

Cottrell, W. O., Keel, M. K., Brooks, J. W., Mead, D. G., & Phillips, J. E. (2013). First report of clinical disease associated with canine distemper virus infection in a wild black bear (*Ursus americana*). *Journal of Wildlife Diseases*, *49*(4), 1024-1027. https://doi.org/10.7589/2013-02-027

Ek-Kommonen, C., Rudbäck, E., Anttila, M., Aho, M., & Huovilainen, A. (2003). Canine distemper of vaccine origin in European mink, *Mustela lutreola*—A case report. *Veterinary Microbiology*, *92*(3), 289-293. https://doi.org/10.1016/s0378-1135(02)00361-9

Fischer, C. D. B., Ikuta, N., Canal, C. W., Makiejczuk, A., Allgayer, M. D. C., Cardoso, C. H., Lehmann, F. K., Fonseca, A. S. K., & Lunge, V. R. (2013). Detection and differentiation of field and vaccine strains of canine distemper virus using reverse transcription followed by nested real time PCR (RT-nqPCR) and RFLP analysis. *Journal of Virological Methods*, *194*(1-2), 39-45. https://doi.org/10.1016/j.jviromet.2013.08.002

Frisk, A. L., König, M., Moritz, A., & Baumgärtner, W. (1999). Detection of canine distemper virus nucleoprotein RNA by reverse transcription-PCR using serum, whole blood, and cerebrospinal fluid from dogs with distemper. *Journal of Clinical Microbiology*, *37*(11), 3634-3643. https://doi.org/10.1128/JCM.37.11.3634-3643.1999

Frölich, K., Czupalla, O., Haas, L., Hentschke, J., Dedek, J., & Fickel, J. (2000). Epizootiological investigations of canine distemper virus in free-ranging carnivores from Germany. *Veterinary Microbiology*, *74*(4), 283-292. https://doi.org/10.1016/s0378-1135(00)00192-9

George, A. M., Wille, M., Wang, J., Anderson, K., Cohen, S., Moselen, J., Lee, L. Y. Y., Suen, W. W., Bingham, J., Dalziel, A. E., Whitney, P., Stannard, H., Hurt, A. C., Williams, D. T., Deng, Y.-M., & Barr, I. G. (2022). A novel and highly divergent canine distemper virus lineage causing distemper in ferrets in Australia. *Virology*, *576*, 117-126. https://doi.org/10.1016/j.virol.2022.09.001

Giacinti, J. A., Pearl, D. L., Ojkic, D., Campbell, G. D., & Jardine, C. M. (2021). Genetic characterization of canine distemper virus from wild and domestic animal submissions to diagnostic facilities in Canada. *Preventive Veterinary Medicine, 198*, 105535. https://doi.org/10.1016/j.prevetmed.2021.105535

Gulliver, E., Taylor, H., Eames, M., Chernyavtseva, A., Jauregui, R., Wilson, A., Bestbier, M., O'Connell, J., Buckle, K., & Castillo-Alcala, F. (2025). Investigation of post-vaccinal canine distemper involving the Rockborn-like strain in nine puppies in New Zealand. *New Zealand Veterinary Journal*, 1–10. https://doi.org/10.1080/00480169.2025.2481896

Kadam, R. G., Karikalan, M., Siddappa, C. M., Mahendran, K., Srivastava, G., Rajak, K. K., Bhardwaj, Y., Varshney, R., War, Z. A., Singh, R., Ghosh, M., Beena, V., Pawde, A. M., Singh, K. P., & Sharma, A. K. (2022). Molecular and pathological screening of canine distemper virus in Asiatic lions,

tigers, leopards, snow leopards, clouded leopards, leopard cats, jungle cats, civet cats, fishing cat, and jaguar of different states, India. *Infection, Genetics and Evolution*, *98*, 105211. https://doi.org/10.1016/j.meegid.2022.105211

Keawcharoen, J., Theamboonlers, A., Jantaradsamee, P., Rungsipipat, A., Poovorawan, Y., & Oraveerakul, K. (2004). Nucleotide sequence analysis of nucleocapsid protein gene of canine distemper virus isolates in Thailand. *Veterinary Microbiology*, *105*(2), 137–142. https://doi.org/10.1016/j.vetmic.2004.10.011

Lednicky, J. A., Dubach, J., Kinsel, M. J., Meehan, T. P., Bocchetta, M., Hungerford, L. L., Sarich, N. A., Witecki, K. E., Braid, M. D., Pedrak, C., & Houde, C. M. (2004). Genetically distant American Canine distemper virus lineages have recently caused epizootics with somewhat different characteristics in raccoons living around a large suburban zoo in the USA. *Virology Journal*, *1*(1). https://doi.org/10.1186/1743-422x-1-2

Martella, V., Blixenkrone-Møller, M., Elia, G., Lucente, Cirone, F., Decaro, N., Nielsen, L., Bányai, K., Carmichael, L., & Buonavoglia, C. (2011). Lights and shades on an historical vaccine canine distemper virus, the Rockborn strain. *Vaccine*, *29*(6), 1222–1227. https://doi.org/10.1016/j.vaccine.2010.12.001

Martella, V., Elia, G., Lucente, M. S., Decaro, N., Lorusso, E., Banyai, K., Blixenkrone-Møller, M., Lan, N. T., Yamaguchi, R., Cirone, F., Carmichael, L. E., & Buonavoglia, C. (2007). Genotyping canine distemper virus (CDV) by a hemi-nested multiplex PCR provides a rapid approach for investigation of CDV outbreaks. *Veterinary Microbiology*, *122*(1-2), 32-42. https://doi.org/10.1016/j.vetmic.2007.01.005

Pardo, I. D. R., Johnson, G. C., & Kleiboeker, S. B. (2005). Phylogenetic characterization of canine distemper viruses detected in naturally infected dogs in North America. *Journal of Clinical Microbiology*, *43*(10), 5009-5017. https://doi.org/10.1128/JCM.43.10.5009-5017.2005

Pekkarinen, H. M., Karkamo, V. K., Vainio-Siukola, K. J., Hautaniemi, M. K., Kinnunen, P. M., Gadd, T. K., & Holopainen, R. H. (2023). Post-vaccinal distemper-like disease in two dog litters with confirmed infection of vaccine virus strain. *Comparative Immunology Microbiology and Infectious Diseases*, *105*, 102114. https://doi.org/10.1016/j.cimid.2023.102114

Rätsep, E., & Ojkic, D. (2024). Canine distemper virus infection of vaccinal origin in a 14-week-old puppy. *Journal of Veterinary Diagnostic Investigation*, *36*(2), 287–290. https://doi.org/10.1177/10406387241229436

Rzezutka, A., & Mizak, B. (2003). Sequence analysis of the fragment of the phosphoprotein gene of Polish distemper virus isolates. *Archives of Virology*, *148*(8), 1623–1631. https://doi.org/10.1007/s00705-003-0128-3

Ricci, I., Cersini, A., Manna, G., Marcario, G. A., Conti, R., Brocherel, G., Grifoni, G., Eleni, C., & Scicluna, M. T. (2021). A canine distemper virus retrospective study conducted from 2011 to 2019 in Central Italy (Latium and Tuscany regions). *Viruses*, *13*(2), 272. https://doi.org/10.3390/v13020272

Shi, N., Zhang, L., Yu, X., Zhu, X., Zhang, S., Zhang, D., & Duan, M. (2021). insight into an outbreak of canine distemper virus infection in masked palm civets in China. *Frontiers in Veterinary Science*, *8*, 728238. https://doi.org/10.3389/fvets.2021.728238

Tamukai, K., Minami, S., Kurihara, R., Shimoda, H., Mitsui, I., Maeda, K., & Une, Y. (2020). Molecular evidence for vaccine-induced canine distemper virus and canine adenovirus 2 coinfection in a fennec fox. *Journal of Veterinary Diagnostic Investigation*, *32*(4), 598–603.https://doi.org/10.1177/1040638720934809

Uema, M., Ohashi, K., Wakasa, C., & Kai, C. (2004). Phylogenetic and restriction fragment length polymorphism analyses of hemagglutinin (H) protein of canine distemper virus isolates from domestic dogs in Japan. *Virus Research*, *109*(1), 59–63. https://doi.org/10.1016/j.virusres.2004.10.008

Wilkes, R. P., Sanchez, E., Riley, M. C., & Kennedy, M. A. (2014). Real-time reverse transcription polymerase chain reaction method for detection of *Canine distemper virus* modified live vaccine shedding for differentiation from infection with wild-type strains. *Journal of Veterinary Diagnostic Investigation*, *26*(1), 27-34. https://doi.org/10.1177/1040638713517232

Yuan, C., Liu, W., Wang, Y., Hou, J., Zhang, L., & Wang, G. (2017). Homologous recombination is a force in the evolution of canine distemper virus. *PLoS ONE*, *12*(4), e0175416. https://doi.org/10.1371/journal.pone.0175416
