# Supplementary material for: Tracking the Spatial and Functional Dispersion of Vaccine-Related Canine Distemper Virus Genotypes: Insights from a Global Scoping Review
Source: Viruses. 2025 Jul 27;17(8):1045. doi: 10.3390/v17081045 (PMC12390544; doi:10.3390/v17081045)
Supplement: Supplementary file 1 [file viruses-17-01045-s001.zip › SupFile 3_Table S3_Vaccine genotypes+Post-vacCD.pdf]

***Supplementary Material 3.*** **Table S3.** Reported Canine Distemper due to America-1/Rockborn-like strains. Host species, post-vaccination distemper or infection with a vaccine field strain (Post-vac CD), sequencing and genotype of the strain is showed.

| Authors reference | Host Specie | | Year | Post-vac CD | Sequencing | Genotype |
|---|---|---|---|---|---|---|
| Haig, 1956 | *Canis lupus familiaris* | Domestic dog | 1956 | Yes | No | America-1 |
| Krakowka et al., 1982 | *Canis lupus familiaris* | Domestic dog | 1982 | Yes | No | America-1 |
| McCandlish et al., 1992 | *Canis lupus familiaris* | Domestic dog | 1992 | Yes | No | America-1 |
| Frolich et al., 2000 | *Martes foina* | Stone marten | 2000 | No | Yes | America-1 |
| Frolich et al., 2000 | *Meles meles* | Badger | 2000 | No | Yes | America-1 |
| Frolich et al., 2000 | *Mustela putorius* | Polecat | 2000 | No | Yes | America-1 |
| Frolich et al., 2000 | *Nyctereutes procyonoides* | Raccoon dog | 2000 | No | Yes | America-1 |
| Frolich et al., 2000 | *Vulpes vulpes* | Red fox | 2000 | No | Yes | America-1 |
| Giacinti et al., 2021 | *Mephitis mephitis* | Striped skunk | 2001 | No | Yes | America-1 |
| Giacinti et al., 2021 | *Neovison neovison* | American mink | 2001 | No | Yes | America-1 |
| Giacinti et al., 2021 | *Procyon lotor* | Raccoon | 2001 | No | Yes | America-1 |
| Rzeutka & Mizak, 2003 | *Canis lupus familiaris* | Domestic dog | 2003 | No | Yes | America-1 |
| Ek-kommonen et al., 2003 | *Mustela lutreola* | European mink | 2003 | No | Yes | America-1 |
| Lednicky et al., 2004a | *Procyon lotor* | Raccoon | 2004 | No | Yes | America-1 |
| Keawcharoen et al., 2005 | *Canis lupus familiaris* | Domestic dog | 2005 | No | Yes | America-1 |
| Martella et al., 2007 | *Canis lupus familiaris* | Domestic dog | 2007 | No | Yes | America-1 |
| Fischer et al., 2013 | *Canis lupus familiaris* | Domestic dog | 2013 | No | Yes | America-1 |
| Wilkes et al., 2014 | *Canis lupus familiaris* | Domestic dog | 2014 | No | Yes | America-1 |
| Anis et al., 2018 | *Canis lupus familiaris* | Domestic dog | 2018 | No | Yes | America-1 |
| Ricci et al.,2021 | *Canis lupus familiaris* | Domestic dog | 2021 | No | Yes | America-1 |
| George et al., 2022 | *Mustela putorius* | Polecat | 2022 | No | Yes | America-1 |
| Pekkarinen et al., 2024 | *Canis lupus familiaris* | Domestic dog | 2024 | No | Yes | America-1 |

| | | | | | | |
|---|---|---|---|---|---|---|
| Kadam et al., 2022 | *Canis lupus familiaris* | Indian pariah dog | 2022 | No | Yes | America-1/Asia-3 |
| Kadam et al., 2022 | *Panthera tigris* | Tiger | 2022 | No | Yes | America-1/Asia-3 |
| Hartley et al., 1974 | *Canis lupus familiaris* | Domestic dog | 1974 | Yes | No | Rockborn |
| Carpenter et al., 1976 | *Mustela nigripes* | Black-footed ferret | 1976 | Yes | No | Rockborn |
| Bush et al., 1976 | *Ailurus fulgens* | Red panda | 1976 | No | No | Rockborn |
| Bestetti et al., 1978 | *Canis lupus familiaris* | Domestic dog | 1978 | Yes | No | Rockborn |
| Kazacos et al., 1981 | *Potos fiavus* | Kinkajou | 1981 | Yes | No | Rockborn |
| Halbrooks et al., 1981 | *Urocyon cinereoargenteus* | Gray Fox | 1981 | Yes | No | Rockborn |
| McCormick, 1983 | *Lycaon pictus* | Lycaon | 1983 | Yes | No | Rockborn |
| Thomas-Baker, 1985 | *Chrysocyon brachyurus* | Maned wolf | 1985 | Yes | No | Rockborn |
| Montali et al., 1987 | *Fennecus zerda* | Fennec fox | 1987 | Yes | No | Rockborn |
| Cornwell et al., 1988 | *Canis lupus familiaris* | Domestic dog | 1988 | Yes | No | Rockborn |
| Van Heerden et al., 1989 | *Lycaon pictus* | Lycaon | 1989 | Yes | No | Rockborn |
| McInnes et al., 1992 | *Speothos venaticus* | South American bush dog | 1992 | Yes | No | Rockborn |
| Sutherland-Smith et al., 1997 | *Mustela lutreola* | European mink | 1997 | Yes | No | Rockborn |
| Frisk et al., 1999 | *Canis lupus familiaris* | Domestic dog | 1999 | No | Yes | Rockborn |
| Pardo et al., 2005 | *Canis lupus familiaris* | Domestic dog | 2005 | Yes | Yes | Rockborn |
| Uema et al.,2005 | *Canis lupus familiaris* | Domestic dog | 2005 | Yes | Yes | Rockborn |
| Martella et al., 2011 | *Canis lupus familiaris* | Domestic dog | 2011 | No | Yes | Rockborn |
| Cottrell et al., 2013 | *Ursus americana* | Black bear | 2013 | No | Yes | Rockborn |
| Kennedy et al., 2019 | *Ailurus fulgens* | Red panda | 2019 | No | Yes | Rockborn |
| Tamukai et al., 2020 | *Fennecus zerda* | Fennec fox | 2020 | No | Yes | Rockborn |
| Woodroffe, 2021 | *Lycaon pictus* | Lycaon | 2021 | Yes | No | Rockborn |
| Shi et al., 2021 | *Paguma larvata* | Masked palm civet | 2021 | No | Yes | Rockborn |
| Rätsep & Ojkic, 2024 | *Canis lupus familiaris* | Domestic dog | 2024 | No | Yes | Rockborn |
| Gulliver et al., 2025 | *Canis lupus familiaris* | Domestic dog | 2025 | No | Yes | Rockborn |
| Gill et al., 1988 | *Mustela putorius* | Polecat | 1988 | Yes | No | NA |
| Harder and Osterhaus, 1980 | *Canis lupus familiaris* | Domestic dog | 1997 | No | No | NA |

| | | | | | | |
|---|---|---|---|---|---|---|
| Fairley et al., 2015 | *Canis lupus familiaris* | Domestic dog | 2015 | Yes | No | NA |
| Vandenberghe et al., 2021 | *Canis lupus familiaris* | Domestic dog | 2021 | Yes | No | NA |
| Vergara-Wilson et al., 2021 | *Chrysocyon brachyurus* | Maned wolf | 2021 | Yes | No | NA |

## REFERENCES


Anis, E., Newell, T. K., Dyer, N., & Wilkes, R. P. (2018). Phylogenetic analysis of the wild-type strains of canine distemper virus circulating in the United States. *Virology Journal, 15*(1). https://doi.org/10.1186/s12985-018-1027-2

Bestetti, G., Fatzer, R., & Frankhauser, R. (1978). Encephalitis following vaccination against distemper and infectious hepatitis in the dog. An optical and ultrastructural study. Acta neuropathologica, 43(1-2), 69–75. https://doi.org/10.1007/BF00685000

Bush, M., Montali, R. J., Brownstein, D., James, A. E., Jr, & Appel, M. J. (1976). Vaccine-induced canine distemper in a lesser panda. Journal of the American Veterinary Medical Association, 169(9), 959–960.

Carpenter, J. W., Appel, M. J., Erickson, R. C., & Novilla, M. N. (1976). Fatal vaccine-induced canine distemper virus infection in black-footed ferrets. Journal of the American Veterinary Medical Association, 169(9), 961–964.

Cornwell, H. J., Thompson, H., McCandlish, I. A., Macartney, L., & Nash, A. S. (1988). Encephalitis in dogs associated with a batch of canine distemper (Rockborn) vaccine. The Veterinary record, 122(3), 54–59. https://doi.org/10.1136/vr.122.3.54

Cottrell, W. O., Keel, M. K., Brooks, J. W., Mead, D. G., & Phillips, J. E. (2013). First report of clinical disease associated with canine distemper virus infection in a wild black bear (*Ursus americana*). *Journal of Wildlife Diseases*, *49*(4), 1024-1027. https://doi.org/10.7589/2013-02-027

Ek-Kommonen, C., Rudbäck, E., Anttila, M., Aho, M., & Huovilainen, A. (2003). Canine distemper of vaccine origin in European mink, *Mustela lutreola*—A case report. *Veterinary Microbiology*, *92*(3), 289-293. https://doi.org/10.1016/s0378-1135(02)00361-9

Fairley, R., Knesl, O., Pesavento, P., & Elias, B. (2015). Post-vaccinal distemper encephalitis in two Border Collie cross littermates. New Zealand Veterinary Journal, 63(2), 117–120. https://doi.org/10.1080/00480169.2014.955068

Fischer, C. D. B., Ikuta, N., Canal, C. W., Makiejczuk, A., Allgayer, M. D. C., Cardoso, C. H., Lehmann, F. K., Fonseca, A. S. K., & Lunge, V. R. (2013). Detection and differentiation of field and vaccine strains of canine distemper virus using reverse transcription followed by nested real time PCR (RT-nqPCR) and RFLP analysis. *Journal of Virological Methods*, *194*(1-2), 39-45. https://doi.org/10.1016/j.jviromet.2013.08.002

Frisk, A. L., König, M., Moritz, A., & Baumgärtner, W. (1999). Detection of canine distemper virus nucleoprotein RNA by reverse transcription-PCR using serum, whole blood, and cerebrospinal fluid from dogs with distemper. *Journal of Clinical Microbiology*, *37*(11), 3634-3643. https://doi.org/10.1128/JCM.37.11.3634-3643.1999

Frölich, K., Czupalla, O., Haas, L., Hentschke, J., Dedek, J., & Fickel, J. (2000). Epizootiological investigations of canine distemper virus in free-ranging carnivores from Germany. *Veterinary Microbiology*, *74*(4), 283-292. https://doi.org/10.1016/s0378-1135(00)00192-9

Gill, J. M., Hartley, W. J., & Hodgkinson, N. L. (1988). An outbreak of post-vaccinal suspected distemper-like encephalitis in farmed ferrets (Mustela putorius furo). New Zealand veterinary journal, 36(4), 173–176. https://doi.org/10.1080/00480169.1988.35525

George, A. M., Wille, M., Wang, J., Anderson, K., Cohen, S., Moselen, J., Lee, L. Y. Y., Suen, W. W., Bingham, J., Dalziel, A. E., Whitney, P., Stannard, H., Hurt, A. C., Williams, D. T., Deng, Y.-M., & Barr, I. G. (2022). A novel and highly divergent canine distemper virus lineage causing distemper in ferrets in Australia. *Virology*, *576*, 117-126. https://doi.org/10.1016/j.virol.2022.09.001

Giacinti, J. A., Pearl, D. L., Ojkic, D., Campbell, G. D., & Jardine, C. M. (2021). Genetic characterization of canine distemper virus from wild and domestic animal submissions to diagnostic facilities in Canada. *Preventive Veterinary Medicine, 198*, 105535. https://doi.org/10.1016/j.prevetmed.2021.105535

Gulliver, E., Taylor, H., Eames, M., Chernyavtseva, A., Jauregui, R., Wilson, A., Bestbier, M., O'Connell, J., Buckle, K., & Castillo-Alcala, F. (2025). Investigation of post-vaccinal canine distemper involving the Rockborn-like strain in nine puppies in New Zealand. *New Zealand Veterinary Journal*, 1–10. https://doi.org/10.1080/00480169.2025.2481896

Haig D.A. (1956). Canine distemper-immunisation with avianised virus Onderstepoort. J Vet Res, 27, 19-53

Halbrooks, R. D., Swango, L. J., Schnurrenberger, P. R., Mitchell, F. E., & Hill, E. P. (1981). Response of gray foxes to modified live-virus canine distemper vaccines. Journal of the American Veterinary Medical Association, 179(11), 1170–1174.

Harder, T. C., & Osterhaus, A. D. (1997). Canine distemper virus-a morbillivirus in search of new hosts?. Trends in microbiology, 5(3), 120–124. https://doi.org/10.1016/S0966-842X(97)01010-X

Hartley W. J. (1974). A post-vaccinal inclusion body encephalitis in dogs. Veterinary pathology, 11(4), 301–312. https://doi.org/10.1177/030098587401100403

Kadam, R. G., Karikalan, M., Siddappa, C. M., Mahendran, K., Srivastava, G., Rajak, K. K., Bhardwaj, Y., Varshney, R., War, Z. A., Singh, R., Ghosh, M., Beena, V., Pawde, A. M., Singh, K. P., & Sharma, A. K. (2022). Molecular and pathological screening of canine distemper virus in Asiatic lions, tigers, leopards, snow leopards, clouded leopards, leopard cats, jungle cats, civet cats, fishing cat, and jaguar of different states, India. *Infection, Genetics and Evolution*, *98*, 105211. https://doi.org/10.1016/j.meegid.2022.105211

Kazacos, K. R., Thacker, H. L. Shivaprasad, H. L. & Burger, P. P. (1981). Vaccine-induced distemper in kinkajous. Journal of the American Veterinary Medical Association 179: 1166-1168.

Keawcharoen, J., Theamboonlers, A., Jantaradsamee, P., Rungsipipat, A., Poovorawan, Y., & Oraveerakul, K. (2004). Nucleotide sequence analysis of nucleocapsid protein gene of canine distemper virus isolates in Thailand. *Veterinary Microbiology*, *105*(2), 137–142. https://doi.org/10.1016/j.vetmic.2004.10.011

Kennedy, J. M., Earle, J. A. P., Omar, S., Abdullah, H., Nielsen, O., Roelke-Parker, M. E., & Cosby, S. L. (2019). Canine and Phocine Distemper Viruses: Global Spread and Genetic Basis of Jumping Species Barriers. Viruses, 11(10), 944. https://doi.org/10.3390/v11100944

Krakowka, S., Olsen, R. G., Axthelm, M. K., Rice, J., & Winters, K. (1982). Canine parvovirus infection potentiates canine distemper encephalitis attributable to modified live-virus vaccine. Journal of the American Veterinary Medical Association, 180(2), 137–139.

Lednicky, J. A., Dubach, J., Kinsel, M. J., Meehan, T. P., Bocchetta, M., Hungerford, L. L., Sarich, N. A., Witecki, K. E., Braid, M. D., Pedrak, C., & Houde, C. M. (2004). Genetically distant American Canine distemper virus lineages have recently caused epizootics with somewhat different characteristics in raccoons living around a large suburban zoo in the USA. *Virology Journal*, *1*(1). https://doi.org/10.1186/1743-422x-1-2

Martella, V., Blixenkrone-Møller, M., Elia, G., Lucente, Cirone, F., Decaro, N., Nielsen, L., Bányai, K., Carmichael, L., & Buonavoglia, C. (2011). Lights and shades on an historical vaccine canine distemper virus, the Rockborn strain. *Vaccine*, *29*(6), 1222–1227. https://doi.org/10.1016/j.vaccine.2010.12.001

Martella, V., Elia, G., Lucente, M. S., Decaro, N., Lorusso, E., Banyai, K., Blixenkrone-Møller, M., Lan, N. T., Yamaguchi, R., Cirone, F., Carmichael, L. E., & Buonavoglia, C. (2007). Genotyping canine distemper virus (CDV) by a hemi-nested multiplex PCR provides a rapid approach for investigation of CDV outbreaks. *Veterinary Microbiology*, *122*(1-2), 32-42. https://doi.org/10.1016/j.vetmic.2007.01.005

McCandlish, I. A., Cornwell, H. J., Thompson, H., Nash, A. S., & Lowe, C. M. (1992). Distemper encephalitis in pups after vaccination of the dam. The Veterinary record, 130(2), 27–30. https://doi.org/10.1136/vr.130.2.27

McCormick, A. E. (1983). Canine distemper in African Cape hunting dogs (Lycaon pictus). Possibly vaccine induced. Journal of Zoo Animal Medicine 14: 66-71.

McInnes, E. F., Burroughs, R. E., & Duncan, N. M. (1992). Possible vaccine-induced canine distemper in a South American bush dog (Speothos venaticus). Journal of wildlife diseases, 28(4), 614–617. https://doi.org/10.7589/0090-3558-28.4.614

Montali, R. J., Bartz, C. R. And Bush. M.1987. Canine distemper virus. In Virus infections of carnivores, M. J. Appel (ed). Elsevier Science Publishers, Amsterdam, The Netherlands, p. 441

Pardo, I. D. R., Johnson, G. C., & Kleiboeker, S. B. (2005). Phylogenetic characterization of canine distemper viruses detected in naturally infected dogs in North America. *Journal of Clinical Microbiology*, *43*(10), 5009-5017. https://doi.org/10.1128/JCM.43.10.5009-5017.2005

Pekkarinen, H. M., Karkamo, V. K., Vainio-Siukola, K. J., Hautaniemi, M. K., Kinnunen, P. M., Gadd, T. K., & Holopainen, R. H. (2023). Post-vaccinal distemper-like disease in two dog litters with confirmed infection of vaccine virus strain. *Comparative Immunology Microbiology and Infectious Diseases*, *105*, 102114. https://doi.org/10.1016/j.cimid.2023.102114

Rätsep, E., & Ojkic, D. (2024). Canine distemper virus infection of vaccinal origin in a 14-week-old puppy. *Journal of Veterinary Diagnostic Investigation*, *36*(2), 287–290. https://doi.org/10.1177/10406387241229436

Rzezutka, A., & Mizak, B. (2003). Sequence analysis of the fragment of the phosphoprotein gene of Polish distemper virus isolates. *Archives of Virology*, *148*(8), 1623–1631. https://doi.org/10.1007/s00705-003-0128-3

Ricci, I., Cersini, A., Manna, G., Marcario, G. A., Conti, R., Brocherel, G., Grifoni, G., Eleni, C., & Scicluna, M. T. (2021). A canine distemper virus retrospective study conducted from 2011 to 2019 in Central Italy (Latium and Tuscany regions). *Viruses*, *13*(2), 272. https://doi.org/10.3390/v13020272

Shi, N., Zhang, L., Yu, X., Zhu, X., Zhang, S., Zhang, D., & Duan, M. (2021). insight into an outbreak of canine distemper virus infection in masked palm civets in China. *Frontiers in Veterinary Science*, *8*, 728238. https://doi.org/10.3389/fvets.2021.728238

Sutherland-Smith, M. R., Rideout, B. A., Mikolon, A. B., Appel, M. J., Morris, P. J., Shima, A. L., & Janssen, D. J. (1997). Vaccine-induced canine distemper in European mink, Mustela lutreola. Journal of zoo and wildlife medicine: official publication of the American Association of Zoo Veterinarians, 28(3), 312–318.

Thomas-Baker, B. (1985). Vaccination-induced distemper in maned wolves, vaccination-induced corneal opacity in a maned wolf. Proceedings of the American Association of Zoo Veterinarians. Annual Report. Scottsdale, Arizona, p. 53.

Tamukai, K., Minami, S., Kurihara, R., Shimoda, H., Mitsui, I., Maeda, K., & Une, Y. (2020). Molecular evidence for vaccine-induced canine distemper virus and canine adenovirus 2 coinfection in a fennec fox. *Journal of Veterinary Diagnostic Investigation*, *32*(4), 598–603.https://doi.org/10.1177/1040638720934809

Uema, M., Ohashi, K., Wakasa, C., & Kai, C. (2004). Phylogenetic and restriction fragment length polymorphism analyses of hemagglutinin (H) protein of canine distemper virus isolates from domestic dogs in Japan. *Virus Research*, *109*(1), 59–63. https://doi.org/10.1016/j.virusres.2004.10.008

Vandenberghe, H., Escauriaza, L., Nye, G., Teague, M., & Granger, N. (2021). Postvaccination encephalomyelitis in German pinschers. The Veterinary record, 188(6), 231–232. https://doi.org/10.1002/vetr.333

Van Heerden, J., Bainbridge, N., Burroughs, R. E. J., & Kriek, N. P. J. (1989). Distemper-like disease and encephalitozoonosis in wild dogs (Lycaon pictus). Journal of Wildlife Diseases 25: 70-75.

Vergara-Wilson, V., Hidalgo-Hermoso, E., Sanchez, C. R., Abarca, M. J., Navarro, C., Celis-Diez, S., Soto-Guerrero, P., Diaz-Ayala, N., Zordan, M., Cifuentes-Ramos, F., & Cabello-Stom, J. (2021). Canine Distemper Outbreak by Natural Infection in a Group of Vaccinated Maned Wolves in Captivity. Pathogens (Basel, Switzerland), 10(1), 51. https://doi.org/10.3390/pathogens10010051

Wilkes, R. P., Sanchez, E., Riley, M. C., & Kennedy, M. A. (2014). Real-time reverse transcription polymerase chain reaction method for detection of *Canine distemper virus* modified live vaccine shedding for differentiation from infection with wild-type strains. *Journal of Veterinary Diagnostic Investigation*, *26*(1), 27-34. https://doi.org/10.1177/1040638713517232

Woodroffe R. (2021). Modified live distemper vaccines carry low mortality risk for captive African wild dogs, Lycaon pictus. Journal of zoo and wildlife medicine: official publication of the American Association of Zoo Veterinarians, 52(1), 176–184. https://doi.org/10.1638/2020-0045
