# Supplementary material for: Tracking the Spatial and Functional Dispersion of Vaccine-Related Canine Distemper Virus Genotypes: Insights from a Global Scoping Review
Source: Viruses. 2025 Jul 27;17(8):1045. doi: 10.3390/v17081045 (PMC12390544; doi:10.3390/v17081045)
Supplement: Supplementary file 1 [file viruses-17-01045-s001.zip › SupFile 4-Tables S4_S5_S6.pdf]

Supplementary Material 4

Table S4. Kruskal–Wallis H-tests were applied to evaluate differences in the level of potentiality (ordinal scale from 1 to 7) according to the functional group of the host species, considered either as origin or destination of the connection.

Genotype	H-statistic	*p*-value
America-1 Origin	18.04	0.00043
America-1 Destination	100.93	9.8×10^{22}
Rockborn-like Origin	1.08	0.584
Rockborn-like Destination	2.27	0.3222

Note: The table shows H-statistics and corresponding *p*-values for both genotypes.

Table S5. Mann–Whitney U tests were used to compare the level of potentiality between connections involving at least one vaccinated host and those not involving vaccinated individuals.

Genotype	U-statistic	*p*-value
America-1	3537.5	0.709
Rockborn-like	376.0	0.163

Note: The table reports U-statistics and *p*-values for both genotypes.

Table S6. Mann–Whitney U tests were applied to assess differences in the level of potentiality between intercontinental and intracontinental connections.

Genotype	U-statistic	p-value
America-1	8274.0	0.124
Rockborn-like	612.0	1.61×10^{-8}

Note: The table presents U-statistics and p-values for each genotype.
